# The intestinal microbiome and metabolome discern disease severity in cytotoxic T-lymphocyte-associated protein 4 deficiency

**DOI:** 10.1186/s40168-025-02028-7

**Published:** 2025-02-11

**Authors:** Prabha Chandrasekaran, Máté Krausz, Yu Han, Noriko Mitsuiki, Annemarie Gabrysch, Christina Nöltner, Michele Proietti, Theo Heller, Caroline Grou, Virginie Calderon, Poorani Subramanian, Drew R. Jones, Yik Siu, Clayton Deming, Sean Conlan, Steven M. Holland, Julia A. Segre, Gulbu Uzel, Bodo Grimbacher, Emilia Liana Falcone

**Affiliations:** 1 Laboratory of Clinical Investigation, National Institute on Aging (NIA), Baltimore, MD USA; 2 Center for Chronic Immunodeficiency (CCI), Medical Center-University of Freiburg, Freiburg, Germany; 3 Department of Rheumatology and Clinical Immunology, Medical Center-University of Freiburg, Freiburg, Germany; 4 Faculty of Biology, Albert-Ludwigs-University of Freiburg, Freiburg, Germany; 5 Laboratory of Clinical Immunology and Microbiology, National Institute of Allergy and Infectious Diseases (NIAID), National Institutes of Health (NIH), Bethesda, MD USA; 6 Division of Molecular Genetics and Pathology, Center for Devices and Radiological Health, Food and Drug Administration (FDA), Silver Spring, MD USA; 7 Clinic Department of Rheumatology and Immunology, Hannover Medical School, Hanover, Germany; 8 Translational Hepatology Section, National Institute of Diabetes and Digestive and Kidney Diseases (NIDDK), National Institutes of Health (NIH), Bethesda, MD USA; 9 Bioinformatics Core, Montreal Clinical Research Institute (IRCM), Montreal, QC Canada; 10 Bioinformatics and Computational Biosciences Branch (BCBB), Office of Cyber Infrastructure and Computational Biology (OCICB), National Institute of Allergy and Infectious Diseases (NIAID), National Institutes of Health (NIH), Bethesda, MD USA; 11 Metabolomics Laboratory, New York University Langone, New York, NY USA; 12 National Human Genome Research Institute (NHGRI), National Institutes of Health (NIH), Bethesda, MD USA; 13 DZIF — German Center for Infection Research, Satellite Center, Freiburg, Germany; 14 CIBSS — Centre for Integrative Biological Signaling Studies, Albert-Ludwigs-University of Freiburg, Freiburg, Germany; 15 RESIST — Cluster of Excellence, Hannover Medical School, Satellite Center Freiburg, Freiburg, Germany; 16 Center for Immunity, Inflammation and Infectious Diseases, Montreal Clinical Research Institute (IRCM), Montreal, QC Canada; 17 Department of Medicine, Université de Montréal, Montreal, QC Canada; 18 Department of Microbiology, Infectious Diseases and Immunology, Université de Montréal, Montreal, QC Canada

**Keywords:** CTLA4 deficiency, Common variable immunodeficiency, Disease severity, Microbiome, Metabolome, Dysbiosis, Gastrointestinal inflammation, Immunomodulator, Immune dysregulation, Autoimmunity, Inborn error of immunity

## Abstract

**Background:**

Cytotoxic T-lymphocyte-associated protein 4 deficiency (CTLA4-D) is an inborn error of immunity (IEI) caused by heterozygous mutations, and characterized by immune cell infiltration into the gut and other organs, leading to intestinal disease, immune dysregulation and autoimmunity. While regulatory T-cell dysfunction remains central to CTLA4-D immunopathogenesis, mechanisms driving disease severity and intestinal pathology are unknown but likely involve intestinal dysbiosis. We determined whether the intestinal microbiome and metabolome could distinguish individuals with severe CTLA4-D and identify biomarkers of disease severity.

**Results:**

The genera *Veillonella* and *Streptococcus* emerged as biomarkers that distinguished CTLA4-D from healthy cohorts from both the National Institutes of Health (NIH) Clinical Center, USA (NIH; CTLA-D, *n* = 32; healthy controls, *n* = 16), and a geographically distinct cohort from the Center for Chronic Immunodeficiency (CCI) of the Medical Center - University of Freiburg, Germany (CCI; CTLA4-D, *n* = 25; healthy controls, *n* = 24). Since IEIs in general may be associated with perturbations of the microbiota, a disease control cohort of individuals with common variable immunodeficiency (CVID, *n* = 20) was included to evaluate for a CTLA4-D-specific microbial signature. Despite common IEI-associated microbiome changes, the two bacterial genera retained their specificity as biomarkers for CTLA4-D. We further identified intestinal microbiome and metabolomic signatures that distinguished patients with CTLA4-D having severe vs. mild disease. Microbiome changes were associated with distinct stool metabolomic profiles and predicted changes in metabolic pathways. These differences were impacted by the presence of gastrointestinal manifestations and were partially reversed by treatment with abatacept and/or sirolimus.

**Conclusions:**

Loss of intestinal microbial diversity and dysbiosis causing metabolomic changes was observed in CTLA4-D. Albeit some of these features were shared with CVID, the distinct changes associated with CTLA4-D highlight the fact that IEI-associated microbiome changes likely reflect the underlying immune dysregulation. Identified candidate intestinal microbial and metabolic biomarkers distinguishing individuals with CTLA4-D based on severity should be studied prospectively to determine their predictive value, and investigated as potential therapeutic targets.

Video Abstract

**Supplementary Information:**

The online version contains supplementary material available at 10.1186/s40168-025-02028-7.

## Introduction

Cytotoxic T-lymphocyte-associated protein 4 (CTLA4) is essential to maintain immune homeostasis. It is expressed by T-cells and outcompetes CD28 for its cognate ligands CD80 and CD86 expressed on effector T-cells (T_eff_) and antigen-presenting cells [1]. CTLA4 is therefore critical to regulatory T-cell (T_reg_) functions such as inhibiting T_eff_ cell proliferation and controlling antigen presentation [2], which together help maintain tolerance. Although common *CTLA4* gene variants have been implicated in the predisposition to several autoimmune conditions [3–5], it was not until 2014 that the first cases of CTLA4 deficiency (CTLA4-D) were described as a rare autosomal dominant immune dysregulation disorder caused by novel germline uniallelic mutations [6, 7]. Patients with CTLA4-D can develop multiorgan autoimmunity and lymphoproliferation that occur with variable severity even among carriers of the same mutation within the same family [6, 8, 9]. The disease spectrum includes enteropathy, hypogammaglobulinemia, recurrent respiratory infections, and lymphocytic infiltration of nonlymphoid organs including the skin, brain, lung, gut, liver, and bone marrow, which are observed along with lymphadenopathy, splenomegaly, autoimmune cytopenia, lymphoma, and autoimmune endocrinopathies [10]. Several patients with CTLA4-D have received allogeneic hematopoietic cell transplantation (HCT) with variable success [11].

The CTLA4 signaling pathway is exploited to treat the clinical manifestations of CTLA4-D with immunomodulators such as sirolimus (a.k.a. rapamycin, a mammalian target of rapamycin [mTOR] inhibitor) and/or abatacept (i.e., a CTLA4 mimetic) [9, 12]. In contrast, the monoclonal antibody-mediated blockade of CTLA4 (e.g., ipilimumab) drives T-cell antitumor activity and has constituted a major breakthrough in the treatment of malignancies such as metastatic melanoma [13]. Unfortunately, immune-related adverse events have been associated with anti-CTLA4 antibodies including the following: vitiligo, colitis, hepatitis, endocrinopathies, inflammatory polyneuropathies, myasthenia gravis, and immune-mediated cytopenia [14].

While T_reg_ dysfunction remains central to the immunopathogenesis of CTLA4-D, the factors driving disease penetrance (estimated at 70% [9]) and expressivity are unknown. However, the wide range of inflammatory dysregulation attributed to CTLA4-D highlight the importance of disease modifiers, such as the environment. In fact, the microbiota and its associated metabolome represent key potential environmental drivers of disease severity. Meanwhile, the contribution of the intestinal microbiota in shaping T_reg_ responses both locally and systemically is well-established [15]. Although several key studies have demonstrated the important role of the microbiota [16, 17] and associated metabolites [18, 19] in mediating response to CTLA4 blockers to treat malignancies, where T_reg_ activity is intentionally dampened, no studies to date have evaluated the role of the intestinal microbiota in driving disease severity in individuals with CTLA4-D. We present the first study evaluating the composition of the intestinal microbiome and metabolome in individuals with asymptomatic or mild compared to severe CTLA4-D. We identified specific bacterial taxa and metabolites associated with severe symptomatic disease and enteropathy, and validated our findings in a separate, geographically distinct patient cohort. Further, we elucidated a specific microbial signature that distinguishes patients with CTLA4-D from those with other IEIs such as common variable immunodeficiency (CVID).

## Methods

### Study design and population

Patients were recruited from the National Institutes of Health (NIH) Clinical Center, USA (NIH; total *n* = 48), between December 2013 and August 2019 and from the Center for Chronic Immunodeficiency (CCI) of the Medical Center of the Faculty of Medicine of Freiburg University Treatment Consortium, Germany (CCI; total *n* = 69, i.e., geographically distinct cohort) between October 2015 and August 2018. All patients providing consent for their stool sample were included, and each cohort was analyzed separately. Data and samples from one visit per patient were included in this cross-sectional observational study.

#### NIH cohort

Healthy individuals (*n* = 16) and patients with CTLA4-D (*n* = 32) provided informed consent in accordance with the Declaration of Helsinki and were enrolled into the National Institute of Allergy and Infectious Diseases (NIAID) Institutional Review Board (IRB)-approved protocol 93-I-0119 — Detection and Characterization of Host Defense Defects (ClinicalTrials.gov: NCT00001355), a prospective longitudinal observational cohort study.

#### CCI cohort

Healthy individuals (*n* = 24) and patients with CTLA4-D (*n* = 25) or CVID (*n* = 20) were recruited; patient consent was collected by the treating physicians, and samples were processed at the Uniklinik Freiburg under the ethics-approved protocols 526/14, 295/13_140782, and 60/18.

### Study procedures

#### NIH cohort

Clinical and laboratory data was retrospectively collected from electronic medical records in accordance with our IRB-approved protocol, and all participants completed a self-administered questionnaire which included the numeric rating scale (NRS) [20], the Short Inflammatory Bowel Disease Questionnaire (SIBDQ) [21], and the Patient Simple Clinical Colitis Activity Index (P-SCCAI) [22]. A Clinical Activity Index (CAI) was assigned based on the following criteria: CAI 1 = 0–2 bowel movements (BM)/day, CAI 2 = 3–4 BMs/day, CAI 3 = > 4 BMs/day, and/or the presence of blood or mucus in stool and/or fistulae and/or perianal disease. Stool samples were self-collected in sterile dry tubes and immediately stored at − 80 °C. All fecal samples were evaluated for the presence of occult blood using the Hemoccult II SENSA Single Slides System (Beckman Coulter). Fecal calprotectin was measured using a Calprotectin ELISA Assay Kit (Eagle Biosciences).

#### CCI cohort

Participant clinical and laboratory data, including medication history, were retrospectively collected from the medical charts as per the IRB-approved protocol. Stools were self-collected in tubes containing a stabilizing liquid (STRATEC) which allows for the conservation of samples up to 3 months at room temperature. Upon arrival, stool samples were homogenized, divided into aliquots, and frozen at − 80 °C.

For both the NIH and CCI cohorts, CTLA4-D disease severity was defined according to the “NIH classification.” Patients with CTLA4-D classified as “mild” had no known clinical manifestation of CTLA4-D or had one or more of the following manifestations: hypogammaglobulinemia, dermatological (e.g., vitiligo or psoriasis), and endocrinological (e.g., type 1 diabetes or Hashimoto’s thyroiditis). Patients with any additional manifestation affecting any other organ system were considered either “severe no GI” or “severe GI” depending on whether they also had any gastrointestinal (GI) complications (i.e., diarrhea lasting more than 3 weeks, celiac disease, enteropathy, lymphocytic colitis, inflammatory bowel disease [IBD]). The choice of clinical manifestations distinguishing patients with CTLA4-D with mild vs. severe disease was based on the observation that patients with manifestations beyond those included in the mild classification also had reduced T_reg_ counts.

### Fecal DNA extraction and amplicon sequencing

#### NIH cohort

Fecal DNA was extracted using the PowerSoil DNA Isolation Kit (MO BIO Laboratories), with the addition of Proteinase K addition (Qiagen) and incubation at 65 °C for 10 min prior to bead beating using a TissueLyser II (Qiagen) at 30 Hz for 2 min. DNA was eluted in UltraClean**®** PCR Water (MO BIO Laboratories), and 16S rRNA gene (V4 region) sequencing was performed using primers (515F = GTGCCAGCAGCCGCGGTAA and 806R = GGACTACCAGGGTATCTAAT) that were modified to include a linker sequence, a 12-bp index sequence, and a heterogeneity spacer [23]. PCR was set up with a LA PCR™ Kit (TaKaRa Bio), and libraries were cleaned up (Agencourt AMPure XP Kit; Beckman Coulter), quantified (Quant-IT dsDNA High-Sensitivity Assay Kit; Invitrogen), and sequenced using a 250-bp paired-end sequencing protocol on the MiSeq platform (Illumina Inc.) at the NIH Intramural Sequencing Center.

#### CCI cohort

Microbial DNA was extracted from stool samples using the QIAamp DNA Stool Mini Kit (Qiagen) with the following modifications: stool lysis was performed at 95 °C, and 400 µl of supernatant (instead of 200 µl) was pipetted into 15 μl of proteinase K, to which 400 µl of AL buffer (instead of 200 µl) was added, followed by mixing and incubation at 70 °C for 10 min. 16S rRNA gene (V3–V4 regions) sequencing was performed using the Illumina 16S metagenomic sequencing library protocol (primers: F = TCGTCGGCAGCGTCAGATGTGTATAAGAGACAGCCTACGGGNGGCWGCAG and R = GTCTCGTGGGCTCGGAGATGTGTATAAGAGACAGGACTACHVGGGTATCTAATCC). PCR was set up with 2 × KAPA HiFi HotStart ReadyMix (Roche). PCR products were purified using magnetic beads (Agencourt AMPure XP-Kit; Beckman Coulter). The index PCR was performed with Nextera XT Index Kit v2 Set B and 2 × KAPA HiFi HotStart ReadyMix and quantified on a TapeStation using D1000 High Sensitivity reagents from Agilent Technologies. The library was denatured with NaOH, diluted, mixed with 5% PhiX control, and loaded on a MiSeq platform (Illumina Inc.) for high-throughput sequencing (2 × 300 cycle V3 kit).

### Stool metabolomics

#### Liquid chromatography-mass spectrometry with hybrid metabolomics method

Liquid chromatography (LC)-mass spectrometry (MS) analysis and metabolite extraction were performed on stool samples from the NIH cohort as described [24]. MS analyses were performed by coupling the LC system to a Thermo Q-Exactive HF mass spectrometer operating in heated electrospray ionization mode. The parameters included the following: 30 min duration, 3.5-kV spray voltage, 320 °C capillary temperature, 35 sheath gas rate, 10 aux gas, 100 μA of max spray current, 120,000 resolution with an automatic gain control target of 3e6, a maximum IT of 100 ms, and scan range from 67 to 1000 m/z. Tandem MS spectra used the following: 15,000 resolution, AGC target of 1e5, maximum IT of 50 ms, isolation window of 0.4 m/z, isolation offset of 0.1 m/z, fixed first mass of 50 m/z, and 3-way multiplexed normalized collision energies of 10, 35, and 80. The minimum AGC target was 1e4 with an intensity threshold of 2e5, and all data were acquired in profile mode.

#### Global metabolomics data processing and relative quantification of metabolites

Using an in-house generated Python script, Thermo RAW files were converted to SQLite format to enable downstream peak detection and quantification. MS/MS spectra were searched against the NIST17MS/MS [25, 26], METLIN [27], and respective Decoy spectral library databases [28, 29] applying a 100% false discovery rate. The peak heights for each sample and metabolite were extracted from the sqlite3 files based on the metabolite retention time ranges and accurate masses. Metabolite peaks were extracted based on the theoretical m/z of the expected ion type (e.g., [M + H] +), with a 15 ppm tolerance ± 0.2 min peak apex retention time tolerance within an initial retention time search window of ± 0.25 min. The final peak detection was calculated based on a signal-to-noise ratio of 3 × compared to blank controls with a floor of 10,000 (arbitrary units). The median metabolite intensity of each sample was used to normalize the intensities to account for inter-batch variations.

### Amplicon sequence and metabolomics data analysis

Raw sequence reads were analyzed using QIIME2 (Quantitative Insights Into Microbial Ecology version 2). Sequence quality analysis was performed using FastQC and sample sequence inference with DADA2 v1.10 [30]. The sequences were clustered into operational taxonomic units (OTUs) using an open-reference OTU picking workflow to classify the resulting amplicon sequence variants (ASVs) with the SILVA v138 database and the species identified by SATé-Enabled Phylogenetic Placement. For all analyses, microbial features were filtered based on having at least 0.01% relative abundance and a 20% prevalence filter (that is 20% of values had at least 4 counts). The data was normalized using total sum scaling. The following R packages were used to perform the indicated analyses: Phyloseq for diversity trends, community composition visualizations, and alpha diversity [31] with comparisons using the Kruskal–Wallis test, EdgeR [32] for differential abundance analyses, metacoder [33] for heat tree analyses, and *lefser* version 1.8.0 [34] for linear discriminant analysis (LDA) effect size (LEfSe) analyses to identify microbiome biomarkers [35]. Beta diversity was evaluated by principal coordinates analyses (PCoA) based on the weighted UniFrac distance method, and significance determined by permutational MANOVA (PERMANOVA, 9999 permutations). Predictive functional profiling of the microbial communities was performed using PICRUSt2 [36]. For integrative analyses, MicrobiomeAnalyst 2.0 was used [37]. The heat tree analyses depicting taxonomic differences utilized the median abundance value and the nonparametric Wilcoxon rank-sum test to leverage the hierarchical structure of taxonomic classifications.

Metabolomics data was processed with an in-house pipeline using Metabolyze version 1.0., and *t*-tests were performed using the Python SciPy library (1.1.0) [38, 39]. The R-library DESeq2 (1.24.0) [40] was used to test for significant differences. Enrichment pathway analyses and metadata analyses were performed using the MetaboAnalyst R package [41]. For accurate prediction of biomarkers, the random forest machine learning algorithm was applied using the randomForest package in R by averaging 1000 decision trees [42].

All diversity trends and community composition visualizations were created using the ggplot2 package in R. This work utilized the computational resources of the NIH HPC Biowulf cluster (http://hpc.nih.gov) and the Nephele platform from the NIAID Office of Cyber Infrastructure and Computational Biology (OCICB) (http://nephele.niaid.nih.gov, Bethesda, MD, USA). All sequences, metadata files, and supplementary files associated with the data analysis are available in the National Center for Biotechnology Information Sequence Read Archive (BioProject ID: PRJNA996628; https://www.ncbi.nlm.nih.gov/sra/PRJNA996628).

### Statistical analyses

Statistical analyses of clinical data were performed using GraphPad Prism Version 9.0. Continuous variables were expressed as means with standard deviations (SD) and categorical variables as absolute values with percentages. Unpaired *t*-tests with Welch correction were used for two-group comparisons, and one-way ANOVA with Welch correction was used for multiple comparisons. All tests were two-sided, and *p* < 0.05 was considered significant. The correlogram of clinical and laboratory variables was constructed with corrplot package in R. The R versions 4.1.3 to 4.2.2 were used for data analysis and visualization. Figures were assembled using Adobe Illustrator or Photoshop.

## Results

### Demographic and clinical characteristics

#### NIH cohort

Clinical characteristics of patients with CTLA4-D compared to healthy individuals recruited from the NIH and included in the study are shown in Supplementary Table S1. The mean age of participants was similar between both groups, but a larger proportion of healthy participants was female (75% vs. 37.5%). Most patients with CTLA4-D were classified as severe (78.2%), and while 71.9% had a history of GI manifestations, only 34.4% had active symptoms at the time of visit. The most common manifestations of CTLA4-D included anemia (50%), endocrinopathy (53.1%), pulmonary (53.1%) and/or central nervous system (CNS) complications (53.1%), and hypogammaglobulinemia (50%). Regarding medications taken regularly, 37.5% of participants were on antibiotics, 43.8% were on sirolimus, 37.5% were on systemic steroids, and 21.9% were on abatacept.

Patients with CTLA4-D had C-reactive protein (CRP) levels that were above the upper limit of normal. Compared to healthy individuals, patients with CTLA4-D were more likely to have detectable fecal occult blood and elevated fecal calprotectin levels. They also had significantly worse quality of life (NRS, SIBDQ) and disease activity (CAI, P-SCCAI) scores (Supplementary Table S2). Correlations between demographic and clinical characteristics are shown in Supplementary Fig. S1.

#### CCI cohort

Clinical characteristics of patients with CTLA4-D compared to healthy individuals recruited from the CCI are shown in Supplementary Table S3. Compared to the NIH cohort, participants included in the CCI cohort were slightly older (mid-40 s versus early 30 s), and most patients with CTLA4-D were female (64%). Less patients with CTLA4-D had severe disease (56%) and a history of GI manifestations (56%). However, all patients with a history of GI manifestations had active symptoms at time of visit. The most common manifestations of CTLA4-D included hypogammaglobulinemia (56%), endocrinopathy (52%), dermatological manifestations (52%), pulmonary complications (40%), and cytopenia (40%). Significantly, less patients in the CCI cohort were on active medications at the time of visit with 12% on antibiotics, 12% on systemic steroids, and none on sirolimus or abatacept.

### Impact of CTLA4 deficiency on intestinal microbiome signatures

**Fig. 1:**
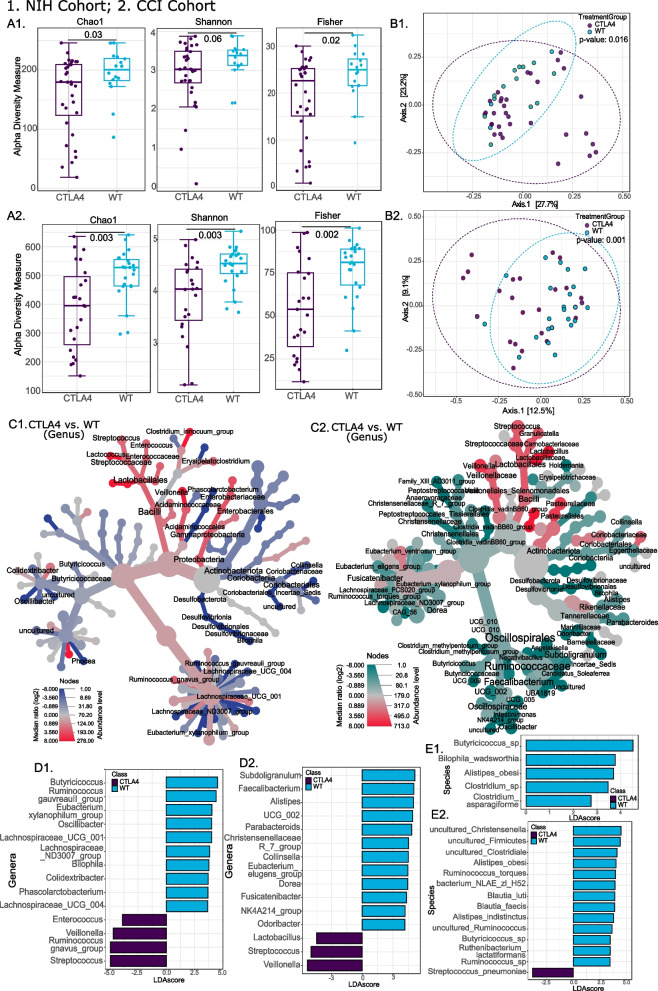
Impact of CTLA4 deficiency on intestinal microbiome signatures. Data for the National Institutes of Health (NIH) Clinical Center and Center for Chronic Immunodeficiency of the Medical Center of the Faculty of Medicine of Freiburg Treatment Consortium (CCI) cohorts are designated by 1 and 2, respectively. Microbiome analyses comparing healthy subjects with patients with CTLA4 deficiency (CTLA4) are shown (NIH cohort: healthy *n* = 16; CTLA4 *n* = 32 and CCI cohort: healthy *n* = 23; CTLA4 *n* = 23). Alpha diversity measures (Chao1, Shannon, and Fisher) with line and whiskers in the box plot representing the median and inter-quartile range, respectively (**A1, A2**). Principal coordinates analysis (PCoA) plot of beta diversity based on unweighted UniFrac distances for the two comparison groups with *p*-values determined by analysis of similarities (ANOSIM) (**B1**, **B2**). Heat tree depicting the significant differential abundances of bacterial genera between CTLA4 and healthy (red = higher abundance; blue or teal = lower abundance) (**C1** and **C2**). Linear discriminant analysis (LDA) score determined by the LDA effect size (LEfSe) analysis showing the defining genera (**D1, D2**) and species (**E1, E2**) for each cohort. *p*-values are provided for each comparison with *p* < 0.05 considered as significant

We examined whether CTLA4-D was associated with changes in alpha diversity (Fig. 1A1, A2) and bacterial composition (Fig. 1B1, B2) in participants from the NIH and CCI cohorts. The richness analysis giving weight to rare species (Chao1) and diversity indices for richness and evenness (Shannon and Fisher) are shown. In both cohorts, patients with CTLA4-D, independent of any other variable including disease severity, had significantly decreased alpha diversity and distinct bacterial composition as measured by beta diversity. The heat trees (Fig. 1C1, C2) and LEfSe graphs show specific genera (Fig. 1D1, D2) and species (Fig. 1E1, E2) that distinguish the intestinal microbiome in patients with CTLA4-D from healthy individuals in each cohort. Notably, samples from patients with CTLA4-D from the NIH cohort were enriched in *Enterococcus* spp., *Veillonella* spp., *Ruminococcus gnavus* group, and *Streptococcus* spp. At the species level, samples were enriched in *Butyricicoccus* sp., *Clostridium* sp., *Bilophila wadsworthia*, *Alistipes obesi*, and *Clostridium asparagiforme*. Compared to healthy individuals, patients with CTLA4-D were depleted in 10 genera including *Butyricicoccus* spp., *Ruminococcus gauvreauii* group, *Eubacterium xylanophilum* group. *Oscillibacter* spp., *Bilophila* spp., *Colidextribacter* spp., *Phascolarctobacterium* spp., and 3 *Lachnospiraceae* spp. (Fig. 1D1, E1, Supplementary Fig. 2A1).

Similar to the NIH cohort, stool samples from patients with CTLA4-D from the CCI cohort were enriched in *Veillonella* spp. and *Streptococcus* spp. while being depleted in *A. obesi* and *Butyricicoccus* spp. Compared to healthy individuals, they were enriched in *Lactobacillus* spp. and *Streptococcus pneumoniae* while being depleted in several other taxa (e.g., *Subdoligranulum* spp., *Faecalibacterium* spp., *Alistipes* spp., *Parabacteroides* spp., *Eubacterium eligens* group, *Fusicatenibacter* spp., *Odoribacter* spp., *Ruminococcus torques*, *Blautia luti*, *Blautia faecis*, a *Ruminococcus* sp., and *Ruthenibacterium lactatiformans*; Fig. 1D2, E2, Supplementary Fig. 2A2). In both cohorts, the phyla Actinobacteriota and Desulfobacterota were significantly less abundant in CTLA4-D (Supplementary Fig. S2B), with the genera *Veillonella* and *Streptococcus* being enriched, as predicted by the generalized linear models to find associations between microbial features and having a *CTLA4* mutation (Supplementary Fig. S2C).

### Impact of disease severity on intestinal microbiome signatures in patients with CTLA4 deficiency

**Fig. 2:**
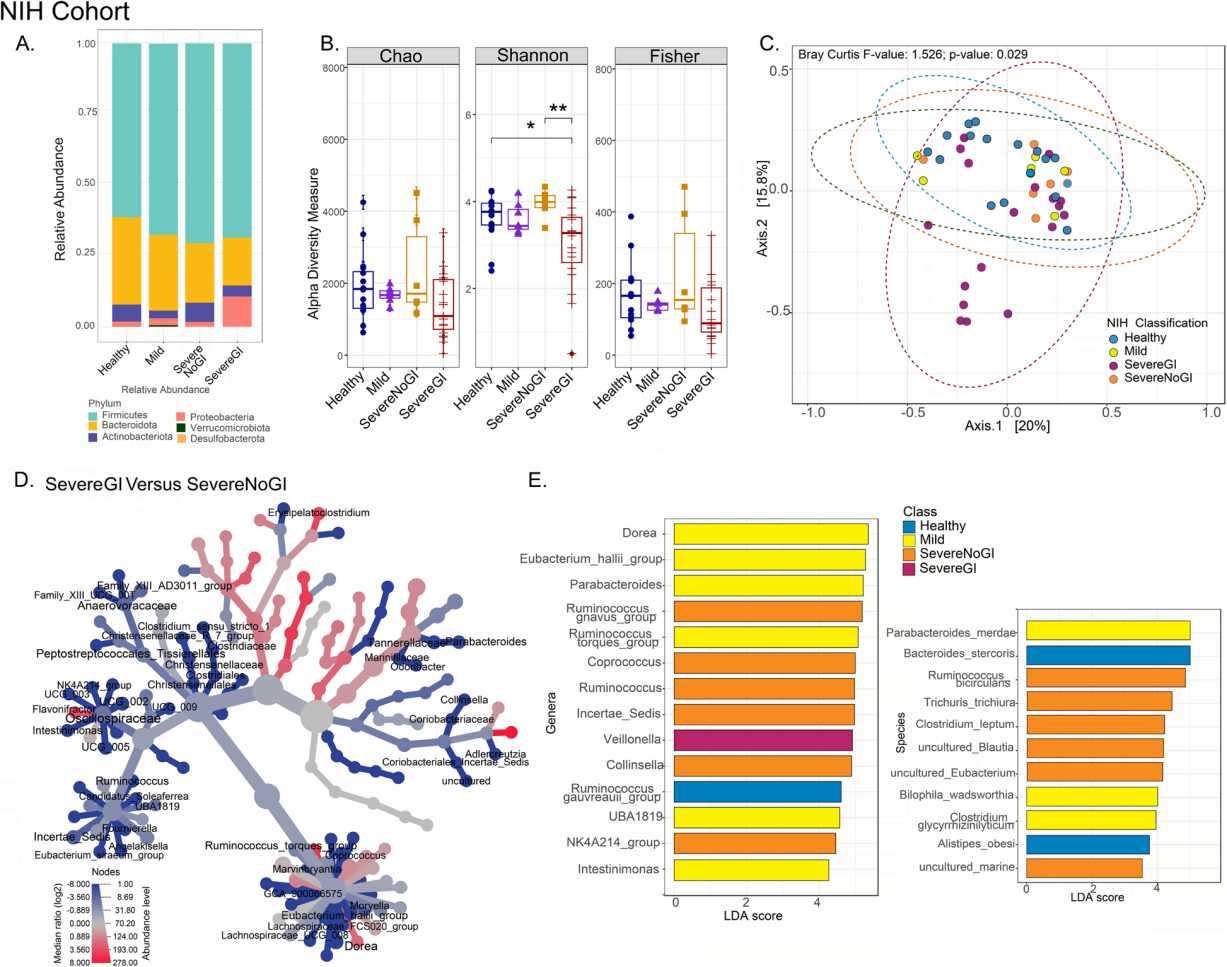
Distinct intestinal microbiome changes associated with CTLA4 deficiency disease severity in the NIH cohort. Comparisons between healthy individuals and patients with different classifications of CTLA4 deficiency based on disease severity are presented for the NIH cohort (healthy *n* = 16, mild *n* = 7, severe without gastrointestinal illness [severe no GI] *n* = 6, severe with GI illness [severe GI] *n* = 19). **A** Relative abundance of phyla. **B** Alpha diversity analyses (*p < 0.05, **p < 0.01). **C** Principal coordinates analysis (PCoA) plot of beta diversity based on the Bray-Curtis metric with *p*-values determined by analysis of similarities (ANOSIM; *p* = 0.029). **D** Heat tree depicting the significant differential abundance (*p*< 0.05) of bacterial genera between severe GI and severe no GI groups (red = higher abundance; blue = lower abundance). Linear discriminant analysis (LDA) score determined by the LDA effect size (LEfSe) analysis showing biomarkers at genus (**E**) and species (**F**) levels

**Fig. 3:**
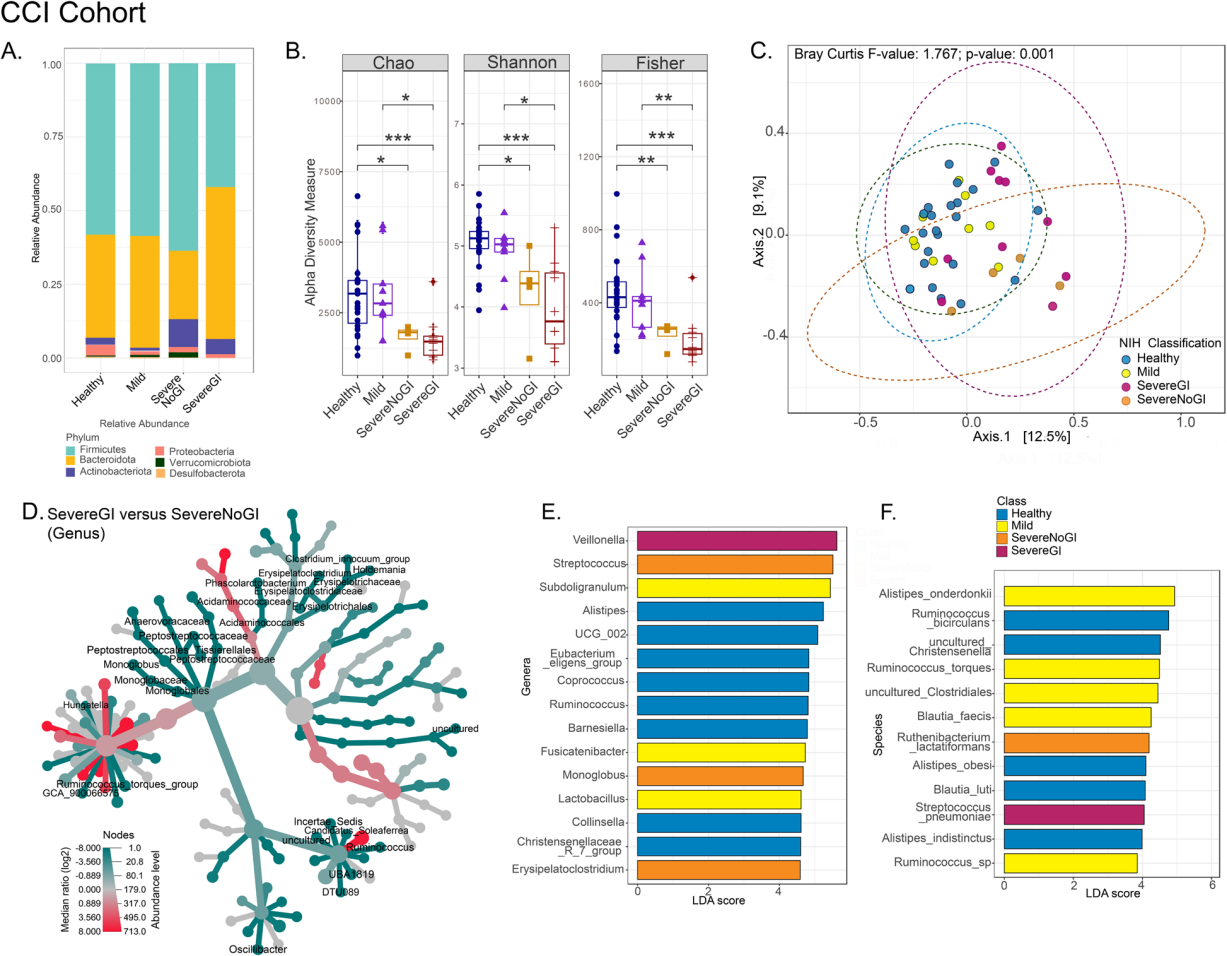
Distinct intestinal microbiome changes associated with CTLA4 deficiency disease severity in the CCI Cohort. Comparisons between healthy individuals and patients with different classifications of CTLA4 deficiency based on disease severity are presented for the CCI cohort (healthy *n* = 23, mild *n* = 9, severe without gastrointestinal illness [severe no GI] *n* = 4, severe with GI illness [severe GI] *n* = 10). **A** Relative abundance of phyla. **B** Alpha diversity analyses (*p < 0.05,**p < 0.01, ***p < 0.001). **C** Principal coordinates analysis (PCoA) plot of beta diversity based on the Bray-Curtis metric with *p*-values determined by analysis of similarities (ANOSIM; *p* = 0.001). **D** Heat tree depicting the significant differential abundance (*p* < 0.05) of bacterial genera between severe GI and severe no GI groups (red = higher abundance; blue = lower abundance). Linear discriminant analysis (LDA) score determined by the LDA effect size (LEfSe) analysis showing biomarkers at genus (**E**) and species (**F**) levels

Since disease severity and GI manifestations appeared to impact intestinal bacterial diversity and composition in both cohorts, we next evaluated each cohort separately to determine whether patients with CTLA4-D had distinct intestinal microbiome signatures based on disease severity (Figs. 2, 3 and Supplementary Fig. S3).

#### NIH cohort

Stool samples from patients with CTLA4-D classified as mild or severe no GI had similar phylum distributions and alpha diversity measures to healthy individuals, whereas patients with severe disease and a history of GI manifestations (severe GI) had reduced intestinal microbiome diversity (Fig. 2A, B) which was characterized by a relative increase in Proteobacteria and decrease in Actinobacteriota and Desulfobacterota (Supplementary Fig. S3A) and a distinct bacterial composition (Fig. 2C). More specifically, samples from patients with CTLA4-D classified as severe GI were enriched in *Veillonella* spp., while samples from patients classified as severe no GI were enriched in *Ruminococcus* spp. (including *R. gnavus* group and *Ruminococcus bicirculans*), *Coprococcus* spp., *Collinsella* spp., and *Clostridium leptum* (Fig. 2D, E, F), indicating potential microbial markers of disease severity. Bacteria from the phyla Verrucomicrobiota and Desulfobacterota were significantly less abundant in severe GI patients compared to mild (Supplementary Fig. S3B). Intestinal microbiome signatures from mild patients were less distinct from healthy individuals (Supplementary Fig. S3C) and were enriched in *Dorea* spp., *Eubacterium hallii*, *Parabacteroides* spp., *Intestinimonas* spp., *Parabacteroides merdae*, *B. wadsworthia*, and *Clostridium glycyrrhizinilyticum* (Fig. 2E, F).

#### CCI cohort

In the CCI cohort, patients classified as mild had similar alpha diversity indices as healthy individuals, whereas patients with severe disease, whether they had a history of GI manifestations or not, had the least diverse microbiome (Fig. 3A, B). Unlike samples from the NIH cohort, samples from the CCI cohort had increased relative abundance of Actinobacteriota but not Proteobacteria (Fig. 3A). However, bacterial community composition was distinct between samples from patients with different degrees of disease severity (Fig. 3C). Similar to the NIH cohort, samples from patients with CTLA4-D and classified as severe GI were enriched in *Veillonella* spp., in addition to *S. pneumoniae*. Samples from patients classified as severe no GI were enriched in *Streptococcus* spp., *Monoglobus* spp., *Erysipelatoclostridium* spp., and *R. lactatiformans*. Samples from patients with mild disease were enriched in several taxa, including *Subdoligranulum* spp*.*, *Fusicatenibacter* spp., *Lactobacillus* spp., *Alistipes onderdonkii*, *B. faecis*, and *R. torques*, which was also enriched in mild patients from the NIH cohort (Fig. 3E, F).

Predictive functional profiling by PICRUSt2 analysis of the microbiome in the NIH cohort comparing stool samples from healthy individuals with patients with CTLA4-D by disease severity showed that a subgroup of patients with severe manifestations of CTLA4-D had microbiome profiles predicted to have significantly increased activity in several pathways involved in menaquinol biosynthesis (Supplementary Fig. S4A). In the CCI cohort, a subgroup of healthy individuals also had microbiome profiles predicted to have increased menaquinol biosynthesis (Supplementary Fig. S4B).

Several patients with CTLA4-D had active GI or a history of GI disease at the time of sample collection. We therefore evaluated intestinal dysbiosis in patients with CTLA4-D from both cohorts based on previous GI disease (Supplementary Fig. S5). As observed in several previous studies, a history of GI disease was associated with changes in phylum distribution and beta diversity (Supplementary Fig. S5A, B). Interestingly, *Veillonella* spp. and *Streptococcus* spp. were no longer identified by LEfSe as markers of a history of GI disease in the NIH cohort, although the log2FC levels were significantly higher in CTLA4-D with a history of GI disease versus no known previous GI history (Supplementary Fig. S5C1, D1). In the CCI cohort, *Veillonella* but not *Streptococcus* spp. were associated with a previous GI history (Supplementary Fig. S5C2, D2).

Microbiome changes together with their predicted functional profiling indicate a clear reduction in microbial diversity associated with increased disease severity, with the *Veillonella* genus emerging as a potential biomarker for the severe GI phenotype in patients with CTLA4-D.

### Microbial signatures specific to CTLA4 deficiency compared to CVID

**Fig. 4:**
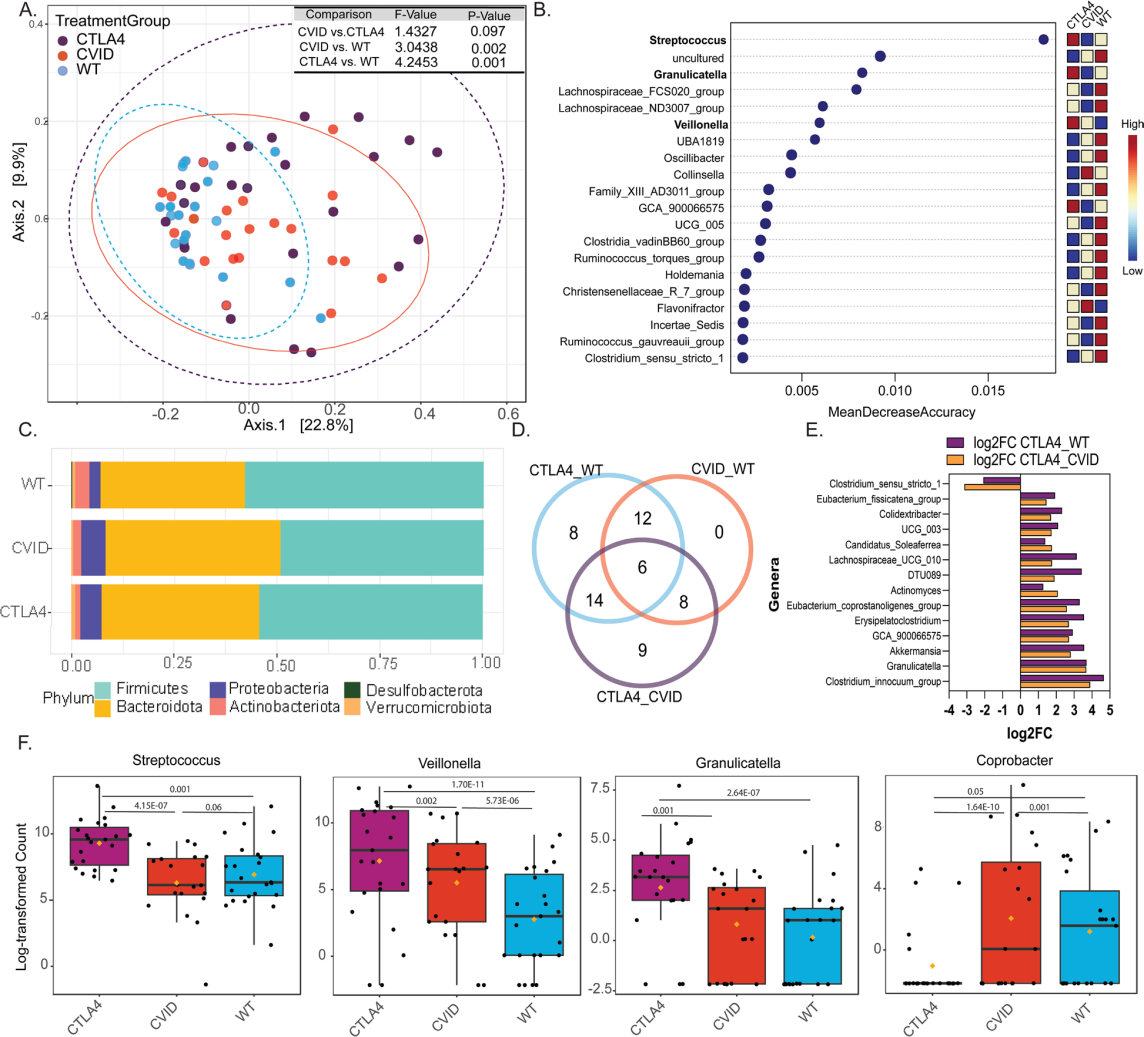
Microbial biomarkers that distinguish CTLA4 deficiency from another inborn error of immunity. Comparisons between healthy (WT) individuals, patients with CTLA4 deficiency (CTLA4), and patients with common variable immunodeficiency (CVID) (**C**) from the CCI cohort (healthy *n* = 23, CTLA4-D *n* = 23, CVID *n* = 20) are shown. **A** Principal coordinates analysis (PCoA) plot of beta diversity based on the Bray–Curtis metric with *p*-values determined by analysis of similarities (ANOSIM). **B** Random forest plots highlighting microbial biomarkers at the genus level (red = high abundance, blue = low abundance). **C** Relative abundance of phyla. **D** Venn diagram of differentially abundant genera between comparison groups (genera are listed in Supplementary Table S4). **E** Genera that are distinct in CTLA4 deficiency. **F** Log-transformed counts of bacterial genera that are potential biomarkers of CTLA4 deficiency

Our high-level analyses revealed intestinal microbiome signatures that readily differentiate patients with CTLA4-D from healthy individuals. We next examined if these biomarkers are specific to CTLA4-D by comparing the CTLA-D intestinal microbiome to that of a cohort of patients with CVID (i.e., an IEI disease control group). The CVID cohort was diverse in terms of clinical presentation, disease severity, and type of immune deficiency. The microbiome signature observed in this cohort therefore represented “non-specific or generic” dysbiosis associated with having an IEI. In other words, the CVID cohort was considered as a negative control to subtract background microbiome changes resulting from immune deficiency. Since CTLA4-D is an autosomal dominant disease that can often involve a CVID phenotype, several clinical and demographic characteristics overlapped between the CTLA4-D and CVID cohorts (Supplementary Table S3). Gut diseases such as colitis (*n* = 3), gastritis (*n* = 5), diarrhea (*n* = 8), intestinal lymphoid nodular hyperplasia (*n* = 2), and GERD (*n* = 3) were observed in patients with CVID. There were no significant differences in beta diversity (Fig. 4A) and phylum distribution (Fig. 4C) between the CTLA4-D and CVID cohorts. However, random forest analyses confirmed the discriminatory power of the genera *Veillonella*, *Streptococcus*, and *Granulicatella*, among others, as biomarkers for CTLA4-D (Fig. 4B). Analysis of genera based on differential abundance fold changes in EdgeR identified 14 genera that can potentially distinguish patients with CTLA4-D from healthy (WT) and patients with CVID (Fig. 4D, F). The common and distinct genera are listed in Supplementary Table S4. The log-transformed counts of *Streptococcus* spp., *Veillonella* spp., and *Granulicatella* spp. showed enrichment specifically in CTLA4-D, while *Coprobacter* spp. were significantly decreased.

### Intestinal metabolomic profiles in patients with CTLA4 deficiency

**Fig. 5:**
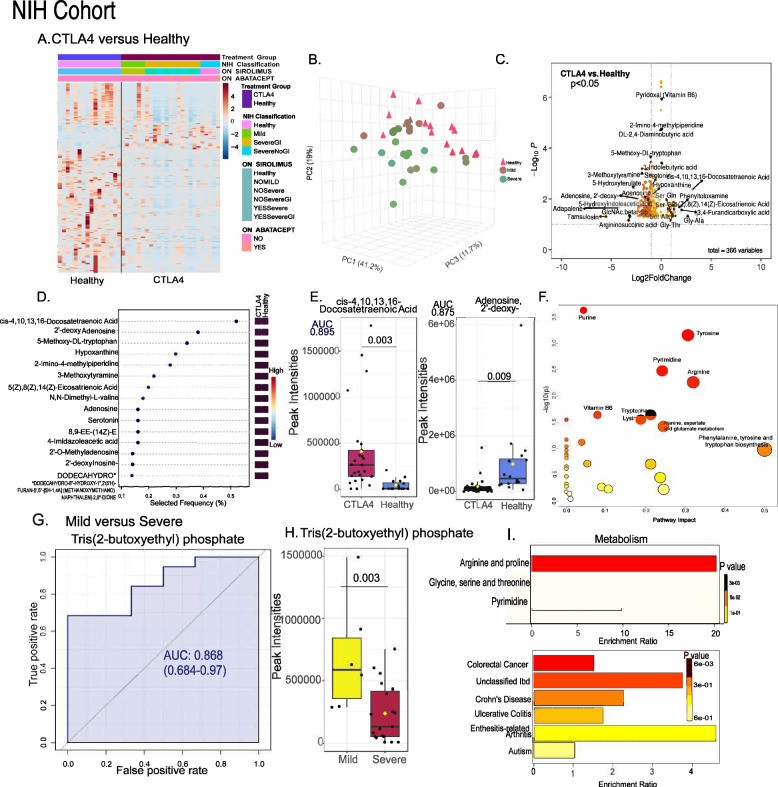
Metabolomic changes in CTLA4 deficiency. Heatmap (**A**), principal coordinates analysis (PCoA) plot (**B**), and volcano plot (**C**) of the metabolomic profiles distinguishing healthy individuals (*n* = 16) from patients with CTLA4 deficiency (CTLA4, *n* = 25). In the heatmap, rows display metabolites, and columns represent samples (blue = decreased, red = increased). The brightness of each color corresponds to the magnitude of the difference when compared with average values. The lines in the volcano plots indicate the significance cutoff for *p*-value (-log10 *p*-value of 1.3013 corresponding to *p* < 0.05) and fold change (FC) (log_2_FC > 2, log_2_FC < −2). All significantly different metabolites are shown. **D** Random forest plots highlighting metabolic biomarkers (red = high intensities, blue = low intensities). **E** Box-whisker plots showing metabolite levels with their area under the curve (AUC) for the receiver operating characteristics (ROC) analysis. **F** Impacted pathways with their respective -log_10_ *p*-values. The size of the bubble is proportional to the size of the *p*-value (the larger the size, the higher the significance). **G** ROC curves are shown for the individual biomarkers along with the AUC value and 95% confidence interval. **H** Distribution of individual values is shown in a box-whisker plot. **I** Enrichment ratio is shown for significant metabolic pathways (top) and associated disease databases (bottom) according to CTLA4 deficiency disease severity. *p*-values are indicated wherever required, and *p* < 0.05 is considered significant

To begin to elucidate the metabolic function associated with the described microbiome signatures, we evaluated the fecal metabolome in patients with CTLA4-D from the NIH cohort (Fig. 5). Fecal samples from patients with mild CTLA4-D had similar metabolomic profiles to healthy individuals (Fig. 5A, B), whereas patients with severe CTLA4-D (with or without GI manifestations) had distinct and less diverse metabolomic profiles compared to healthy and mild groups, reflecting similarities with microbiome profiles. Notably, samples from patients with CTLA4-D had significantly increased levels of cis-4,10,13,16-docosatetraenoic acid (*p* = 0.003) and decreased levels of adenosine, 2′-deoxy (*p* = 0.009) compared to healthy individuals (Fig. 5C, E), and both metabolites were predicted to be metabolic makers for CTLA4-D by random forest analysis (Fig. 5D). Pathway analysis of metabolic profiles highlighted many of the amino acid metabolism pathways including phenylalanine, tyrosine, and tryptophan biosynthesis as being impacted in patients with CTLA4-D (Fig. 5F). To identify markers of severity, mild and severe patients were compared. The area under receiver operating characteristic curve (AUROC) identified reduced tris (2-butoxyethyl) phosphate levels (Fig. 5G, H) in patients with severe disease. The fecal metabolomic profiles clearly demonstrated that amino acid and nucleotide metabolic pathways were highly affected in severe compared to mild patients (Fig. 5I, upper graph) with tris(2-butoxyethyl) phosphate potentially being a marker for progression to severe disease. Moreover, the observed metabolic profiles have been associated with other inflammatory and/or autoimmune pathologies, including colorectal cancer, inflammatory bowel disease, and enthesitis-related arthritis (Fig. 5I, lower graph).

### Treatment with inhibitors of T-cell- or immune cell-mediated inflammation was associated with improved intestinal dysbiosis in patients with CTLA4 deficiency

To determine other major drivers of intestinal microbiome changes, we compared measures of alpha and beta diversity based on the presence or absence of GI manifestations and current treatments in the CTLA4-D groups from NIH and CCI (Supplementary Fig. S6A, B). Age and sex had modest effects on microbial diversity and community structure in both cohorts, while any history of or active GI disease significantly impacted both alpha and beta diversity in the NIH cohort and had only a mild impact on beta diversity in the CCI cohort.

Patients with CTLA4-D and severe manifestations are often treated with immunomodulators, particularly abatacept (i.e., a CTLA4 mimetic) and/or sirolimus (a.k.a. rapamycin, mTOR inhibitor) (Supplementary Fig. S7), which may alter T_reg_ function in patients with CTLA4-D and by extension have an important impact on the microbiota [43]. As previously mentioned, an important difference between the NIH and CCI cohorts was the fact that 56% of patients with severe disease in the NIH cohort were on sirolimus and/or abatacept compared to none in the CCI cohort (Supplementary Tables S1 and S3). We further observed that having a history of GI manifestations seemed to be one of the main factors impacting microbiome signature in the NIH cohort (Supplementary Fig. S5), whereas in the CCI cohort, having severe disease independent of GI manifestations altered the microbiota. We therefore hypothesized that treatment with sirolimus and/or abatacept improved intestinal dysbiosis in the NIH cohort, thereby resulting in GI manifestations having more impact on the intestinal microbiome than disease severity. In contrast, the lack of treatment with abatacept and/or sirolimus in the CCI cohort further highlighted the impact of CTLA4-D disease severity on the intestinal microbiome. Thus, we evaluated the impact of treatment with sirolimus and/or abatacept on the microbiome in patients with CTLA4-D from the NIH cohort.

**Fig. 6:**
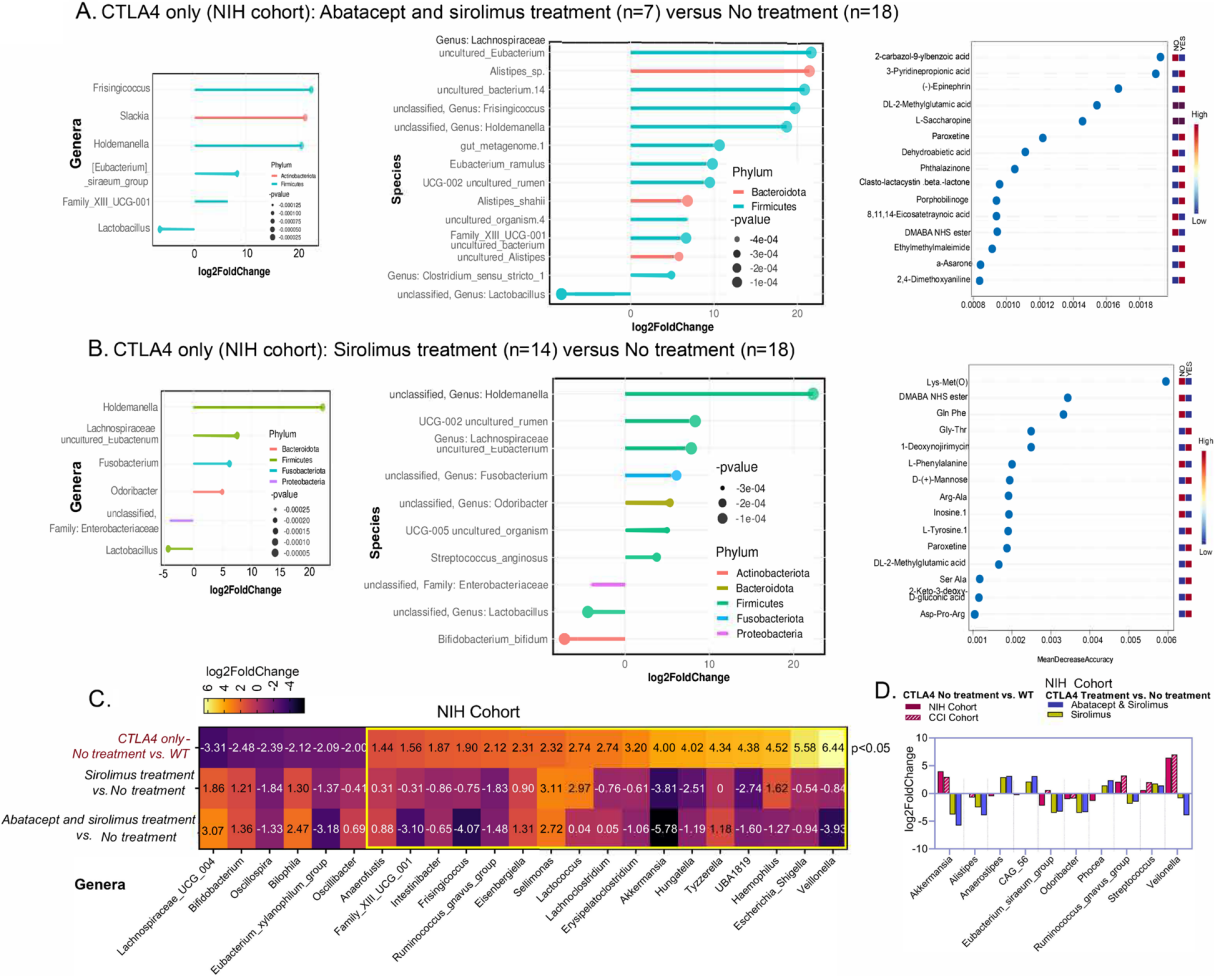
Effects of abatacept and sirolimus on restoring the intestinal microbiota in patients with CTLA4 deficiency. **A**, **B** Linear discriminant analysis (LDA) score determined by LDA effect size (LEfSe) analysis showing bacterial biomarkers at the genus (left) and species levels (middle), and random forest plots (right) showing fecal metabolic biomarkers (red = high abundance, blue = low abundance) for treated compared to untreated patients with CTLA4 deficiency (CTLA4). **C** Heatmap showing log_2_ fold change (FC) of distinguishing bacterial genera for the listed comparisons (i.e., untreated patients with CTLA4 deficiency vs. healthy individuals (WT), sirolimus-treated vs. untreated patients with CTLA4 deficiency, abatacept- and sirolimus-treated vs. untreated patients with CTLA4 deficiency). **D** Bar graph showing log_2_FC of bacterial genera that were restored in treated groups compared to untreated patients with CTLA4 deficiency

When comparing patients that were on sirolimus and abatacept, which were mostly patients with CTLA4-D classified as severe GI, to other CTLA4-D patients who were not on these drugs, we observed significant increases in certain taxa including *Holdemanella* spp., *Frisingicoccus* spp., *Slackia* spp. and *Alistipes* spp., and decreased abundance of *Lactobacillus* spp. (Fig. 6A). Patients on sirolimus (with or without abatacept) had increased relative abundance of *Holdemanella* spp., *Streptococcus anginosus*, unidentified species from the genera *Odoribacter* and *Fusobacterium*, and uncultured *Eubacterium* from the Lachnospiraceae family (Fig. 6B). Random forest analyses identified metabolites predicted to be significantly associated with the administration of abatacept and sirolimus (Fig. 6A) or sirolimus (Fig. 6B). Both drugs increased levels of DL-2-methyl glutamic acid, a derivative of glutamic acid, and reduced 4-(dimethylamino)-benzoic acid, 2,5-dioxo-1-pyrrolidinyl ester (DMABA NHS ester), a synthetic derivative of the naturally occurring amino acid, N-dimethylamino-2-butanol (DMAB). Sirolimus treatment was clearly associated with changes in the metabolism of amino acids identified to be altered in CTLA4-D. Each treatment was associated with clinical improvement and restoration of changes in abundance of certain bacteria and metabolites related to amino acid metabolism in patients with CTLA4-D.

The therapeutic effect of these drugs on CTLA4-D-induced intestinal dysbiosis was further evaluated by comparing the differential abundance of genera in untreated patients (i.e., no treatment with abatacept or sirolimus) with CTLA4-D with either 1) healthy individuals (WT), 2) patients with CTLA4-D treated with sirolimus, or 3) patients with CTLA4-D treated with sirolimus and abatacept (Fig. 6C). Of the 6 genera that were decreased in CTLA4-D patients compared to healthy individuals, 3 were restored with treatment, including a 9-fold (i.e., 3.07 log_2_ fold) increase in Lachnospiraceae spp. Several of the bacterial genera found to be abundant in untreated patients with CTLA4-D were significantly reduced with immunomodulator treatment. The highest treatment-driven differences were noted for *Akkermansia* spp., *Frisingicoccus* spp., and *Veillonella* spp. Interestingly, the genera *Akkermansia*, *R. gnavus* group, and *Veillonella,* which were found to be enriched in untreated patients with CTLA4-D from both the NIH and CCI cohorts (red bars; Fig. 6D), were reduced > 2.5 log_2_ fold in patients with CTLA4-D treated with an immunomodulator from the NIH cohort (yellow and blue bars; Fig. 6D). Treatment with both sirolimus and abatacept showed better reversal of intestinal dysbiosis compared to treatment with sirolimus alone (Fig. 6C, D).

## Discussion

To our knowledge, this is the first study to date evaluating the intestinal microbiome and metabolome in patients with CTLA4-D. Our study is also novel insofar as it identified changes in the intestinal microbiome and metabolome that distinguished patients with severe disease, and identified both common and distinct microbial signatures of CTLA4-D compared to other IEIs such as CVID. Our study also included a second cohort of patients recruited from a different continent, which allowed us to identify a core group of bacterial taxa that were altered in the context of severe disease in both cohorts. Moreover, we identified that treatment with sirolimus and/or abatacept improves clinical disease in association with the reduction of certain bacterial taxa, and metabolic changes.

Immune checkpoint inhibitors such as CTLA4 monoclonal antibodies (e.g., ipilimumab, tremelimumab) are increasingly used to treat various cancers. Despite their therapeutic advantages, CTLA4 blockers can result in life-threating immune-related adverse events [44] that are associated with dysregulation of GI immunity [45]. Meanwhile, *Ctla4*^*−/−*^ mice exhibit diffuse infiltration of multiple organs with inflammatory immune cells and peripheral T-cell proliferation resulting in fatal multiorgan tissue destruction [46, 47]. Although the clinical presentation of patients with CTLA4-D shares some similarities with the side effects of treatment with CTLA4 inhibitors, and immune responses observed in *Ctla4*^*−/−*^ mice (e.g., GI illness, lymphoproliferation, tissue destruction etc.), the manifestations of CTLA4-D in both study cohorts ranged from mild to severe even within the same family [6, 9], suggesting that an extrinsic factor may be driving disease severity.

A potential role for the microbiome as a driver of disease severity in CTLA4-D is not only presented in this study but also supported by studies reporting changes in the structure and function of the microbiome with CTLA4 blockade (either alone or in combination with other immune checkpoint inhibitors) and their association with response to therapy [48–51]. Indeed, studies have demonstrated that the efficacy of CTLA4 blockade partly depends on the host microbiota by showing, for example, that fecal microbiota transplantation (FMT) from patients with multiple myeloma treated with CTLA4 blockade into germ-free mice reduced tumor size in a manner that depended on the presence of distinct *Bacteroides* species [16]. Similarly, two clinical trials demonstrated that FMT can promote therapeutic response in patients with immunotherapy-refractory melanoma [52, 53]. In these studies, FMT promoted favorable changes in proteomic and metabolomic signatures, immune cell infiltrates and CD8 + T-cell activation, and decreased the frequency of interleukin-8-expressing myeloid cells. Other studies have underlined how certain changes in the intestinal microbiota may predispose to colitis associated with anti-CTLA4 and/or anti-PD1 therapy [54–57]. Meanwhile, treatment with certain probiotics [58–60] or FMT [61–63] may help treat immune checkpoint inhibitor-associated colitis by altering the proportion and function of T_regs_ and other immune cells in the intestinal mucosa in addition to reconstituting the gut microbiota. Our study established the direct impact of CTLA4 on intestinal health through alterations in the microbiome and associated metabolome by demonstrating that immune dysregulation resulting from CTLA4-D leads to altered intestinal homeostasis with reduced bacterial alpha diversity and significant changes in beta diversity in comparison to healthy; the degree of impact likely agree with clinical severity and possibly gene penetrance.

CTLA4-D was associated with significantly reduced relative abundances of Actinobacteriota and Desulfobacterota, and increased Proteobacteria compared to healthy individuals. The genera *Veillonella* and *Streptococcus* were significantly enriched in CTLA4-D subjects compared to healthy individuals in both cohorts. There was also a large number of bacterial genera reported to have beneficial intestinal functions that were reduced in patients with CTLA4-D compared to healthy individuals, which included but was not limited to *Oscillibacter* (i.e., has positive regulatory effects in obesity and chronic inflammation [64]), *Odoribacter* (i.e., limits colonic inflammation when transplanted for ulcerative colitis [65]), *Dorea* (Lachnospiraceae family, associated with positive response to CTLA4 blockade [66]), *Collinsella* (i.e., produces ursodeoxycholate and mitigates COVID-19 [67]), and *Butyricicoccus* (i.e., produces the short-chain fatty acid (SCFA) butyrate which has anti-inflammatory properties and helps maintain intestinal barrier integrity [68, 69]). In patients with severe GI CTLA4-D, altered abundances of Firmicutes, Bacteroidota, and Proteobacteria were observed in both cohorts. Moreover, samples from patients with severe GI CTLA4-D were enriched with members of the *R. torques* group but depleted in *Erysipelatoclostridium*, Peptostreptococcaceae, and *Clostridium* spp. The genus *Veillonella* was again significantly enriched in severe GI patients from both cohorts, while the species *S. pneumoniae* was only enriched in severe GI patients from the CCI cohort. *Veillonella* was also enriched in patients with CTLA4-D and any previous history of GI disease. However, the temporal relationship needs to be studied in detail to understand if enrichment with *Veillonella* drives GI severity or vice versa.

While deeper metagenomic sequencing will be required to identify the specific species or strains of *Veillonella* that are enriched in patients with severe GI CTLA4-D, several studies have identified *Veillonella* spp*.* and specifically *Veillonella parvula*, as markers of intestinal dysbiosis and drivers of several inflammatory conditions when in overabundance. These include IBD, Hirschsprung’s disease-associated enterocolitis, primary sclerosing cholangitis, and systemic lupus erythematosus [70–72]. We further reported enrichment with *Holdemanella* spp. in association with abatacept and/or sirolimus treatment. Interestingly, *Holdemanella biformis* (the human homolog of *Faecalibaculum rodentium* found in rodents) may protect against intestinal tumorigenesis [73] and is known to produce the SCFA butyrate, which helps mitigate inflammation and improve T_reg_ responses [74–76]. Treatment with abatacept and/or sirolimus was associated with the improvement of intestinal dysbiosis as evidenced by increased diversity in the CTLA4-D-treated group and the absence of significant differences in alpha and beta diversity compared to healthy individuals. Combination therapy with abatacept and sirolimus was effective in reducing *Veillonella* spp. and increasing *Anaerostipes*, a bacterial genus associated with a beneficial role in gut health through its conversion of dietary inositol into the SCFAs propionate and acetate [77].

Reduced alpha and beta diversity associated with increased levels of circulating lipopolysaccharide (LPS) has been reported in other IEIs such as CVID [78]. Notably, patients with CVID and non-infectious complications often have worse clinical outcomes associated with intestinal dysbiosis [79]. Similar findings were observed in patients with CTLA4-D, leading us to perform comparative analyses between CVID and CTLA4-D to confirm the specificity of the microbiome signatures identified in CTLA4-D. Like in CVID, the alpha diversity was reduced in patients with CTLA4-D compared to healthy individuals with shared changes in the microbial composition. Nevertheless, the CTLA4-D microbiome signature was distinct from that observed in CVID with significantly increased abundance of *Veillonella*, *Streptococcus*, and *Granulicatella* and almost undetectable levels of *Coprobacter*. Our observations highlight that although reduced microbial diversity can be observed in other IEIs, specific microbial changes can distinguish certain IEIs while possibly reflecting the underlying immune dysregulation.

T-cell receptor engagement with the major histocompatibility complex accompanied by a costimulatory signal from CD28 leads to T-cell activation, proliferation, and differentiation, all of which are coupled with increased metabolic requirements for substrates such as glucose, and amino acids such as glutamine (Gln), alanine (Ala), serine (Ser), leucine (Leu), methionine (Met), arginine (Arg), cysteine (Cys), and cystine (Cys-Cys) [80]. In patients with CTLA4-D, these metabolic pathways were significantly associated with increased disease severity. It is plausible that microbiome changes directly impacted intestinal metabolism, which by extension, contributed to increased T-cell activity in patients with CTLA4-D. Future studies should address how microbiome-mediated changes in amino acid, glucose, and nucleotide metabolism in the gut impact T-cell phenotypes and function.

*Streptococcus* spp. and *Veillonella* spp. can be found in the human small intestine, and their co-occurrence has been demonstrated in various studies [81]. The small intestinal microbiota, including streptococci, is involved in the conversion of simple carbohydrates to lactic acids, which is a preferred substrate for *Veillonella* [82]. The co-occurrence of these genera together with *Granulicatella* might depend on their potential for metabolic interactions as shown in the oral cavity [83]. These metabolic interactions could be a key determining factor in the enrichment of certain microbial species, leading to alterations in the intestinal metabolome, which can then impact host immune responses. Interestingly, the predictive pathway analysis associated with microbiome changes in CTLA4-D was characterized by substantial changes in menaquinone (i.e., vitamin K2) biosynthesis, especially in patients with severe disease. Interestingly, vitamin K deficiency has been observed in chronic GI disorders and IBD [84]. However, the underlying mechanisms remain to be elucidated. It is plausible that CTLA4-D-associated intestinal dysbiosis can impactvitamin K2 levels, and its repletion through supplementation may improve both intestinal dysbiosis and inflammation [85].

Undoubtedly, 16S rRNA gene sequencing analysis of the intestinal microbiome combined with metabolomics in patients with CTLA4-D uncovered several interesting new findings. However, future studies should include metagenomic sequencing analysis of fecal and intestinal mucosal samples to identify specific bacterial strains distinguishing patients with CTLA4-D with severe disease from those with asymptomatic or mild disease. Longitudinal studies are also needed to identify microbial biomarkers that will predict who will develop severe disease as these patients may preemptively be treated with immunomodulators or in some cases HCT. Together, these studies would lead to the identification of more specific biomarkers and novel therapeutic strategies for CTLA4-D, including FMT or probiotics, while providing additional insight into the relationship between immune dysregulation, microbial metabolism, and disease penetrance.

## Conclusions

Individuals with clinically severe CTLA4-D have distinct intestinal microbiome and metabolome signatures that are impacted by GI pathology and improve with immunomodulator treatment. Our study identified candidate microbes and metabolites to be further investigated as biomarkers that may predict progression to severe CTLA4-D and/or as novel therapeutic targets.

## Supplementary Information

Supplementary Material 1: Figure S1. Correlations between clinical parameters associated with gastrointestinal (GI) manifestations in patients with CTLA4 deficiency. Correlogram for clinical parameters in patients with CTLA4 deficiency. Circle values=coefficient of correlation (r value); circle size=strength of significance (red=positive correlation, blue=negative correlation, blank=no significant correlation). All presented r values have *p<*0.05. Figure S2. Alterations in phylum and genus abundances in patients with CTLA4 deficiency from NIH and CCI cohorts. (A) Heatmap of components of the core microbiome at the genus level that are detected in high fractions in CTLA4 deficiency groups (20% of the sample prevalence cut-off) (yellow = low prevalence, purple = high prevalence) (A1 = NIH cohort; A2 = CCI cohort). The generalized linear models (GLM) to find associations between microbial features and CTLA4 deficiency identified the phyla (B) and genera (C) that are significantly different in CTLA4 deficiency groups compared to healthy individuals. All comparisons for the genera are significant with *p<*0.05, unless a *p*-value is shown. Figure S3. Phylum- and genus-level differences in CTLA4 deficiency in the NIH cohort. Comparisons are provided for groups of patients with CTLA4 deficiency from the NIH cohort with different degrees of disease severity (Healthy *n=*16, Mild *n=*7, Severe No GI *n=* 6, Severe GI *n=* 19). (A) Box and violin plots indicating phylum abundances in each group. The name of the phylum is indicated in the top of each panel with the *p*-values for each comparison shown in the graph. Wherever the *p*-value is <0.05, the significance is marked with an asterisk (* = *p<*0.05, **=*p<*0.01, ***p<0.001). (B) Heat trees depicting the significant differential abundances (*p<*0.05) of bacterial genera between patients with CTLA4 deficiency with Severe versus Mild disease, and (C) Mild disease versus Healthy (red = higher abundance; blue = lower abundance). Figure S4. Distinct functional profiles in patients with CTLA4 deficiency. Heatmap of significantly different functional profiles inferred by PICRUSt2 performed to identify the pathways associated with changes in the microbiome in CTLA4 deficiency (blue represents higher abundance and yellow represents lower abundance). The relative abundance normalized to a Z-score was used to generate the heatmaps. Pathway comparisons of the CTLA4 deficiency group with Healthy are shown for the NIH cohort (A) (Healthy *n=*16, Mild *n=*7, Severe No GI *n=*6, Severe GI *n=*19) and the CCI cohort (B) (Healthy *n=*23, Mild *n=*9, Severe No GI *n=*4, Severe GI *n=*10). Figure S5. Phylum- and genus-level differences in patients with CTLA4 deficiency and a history of gastrointestinal (GI) manifestations from the NIH and CCI cohorts. Comparisons are provided for groups of patients with CTLA4 deficiency (CTLA4-D) from the NIH (A1, B1, C1, D1) and CCI cohorts (A2, B2, C2, D2) with a history of GI disease (NIH Cohort: No GI history *n=*9, YES GI history *n=*23; CCI Cohort: No GI history *n=*11, YES GI history *n=*14). (A) Phylum distribution and (B) principal coordinates analysis (PCoA) plot of beta diversity based on the Bray Curtis metric with *p*-values determined by analysis of similarities (ANOSIM). (C) Differentially abundant genera and (D) linear discriminant analysis (LDA) scores determined by the LDA effect size (LEfSe) analysis showing biomarkers at the genus level. Box plots of log-transformed counts for select genera are shown on the right. Figure S6. Differences in alpha and beta diversity measures in NIH and CCI cohorts based on clinical characteristics in the CTLA4 deficiency groups. Heat table with *p*-values listed for comparisons of alpha (Chao1, Shannon, Simpson, Fisher) and beta (ANOSIM, Permanova, Permdisp) diversity indices based on characteristics of patients with CTLA4 deficiency in the NIH (A) and CCI (B) cohorts. The darker the pink color, the higher the significance. Orange to yellow shades represent *p*-values between 0.05 and 0.08 (the lighter the color, the lesser the significance). ANOSIM tests whether distances between are greater than within groups. Permanova tests whether distances differ between groups. Permdisp calculates an F-statistic to assess whether the dispersions between groups is significant. Figure S7. Mechanism of inhibition of T-cell inflammation by abatacept (CTLA4 fusion protein), and sirolimus (mTOR inhibitor). Abatacept, a fusion protein of the Fc fragment of IgG1 and extracellular domain of CTLA4, binds to CD80/86 (B7.1. / B.7.2) in antigen presenting cells (APC) or B-cells, and prevents interaction with the CD28 receptor. Thus, it blocks the secondary signal required for immune cell activation following T-cell receptor (TCR) and Major Histocompatibility Complex (MHC)-II binding, thereby reducing T-cell activation and infiltration (left). The mammalian target of Rapamycin complexes (mTORC1 and mTORC2) are activated upon T-cell activation, growth factor or nutrient signaling, and trigger the 4EPB1 (Eukaryotic translation initiation factor 4E [eIF4E]-binding protein 1) and S6 kinase 1 (S6K1) pathways, and protein kinases Akt and PKCa involved in T-cell transcription, protein synthesis and cell cycle regulation. Sirolimus forms a complex with FKBP12 (FK506-binding protein), targets mTORC1 and mTORC2, and inhibits downstream pathways and associated functions (right).

Supplementary Material 2: Table S1. Demographic and clinical characteristics of participants from the NIH cohort. Table S2. Clinical scores and laboratory test results in participants from the NIH cohort. Table S3. Demographic and clinical characteristics of participants from the CCI cohort. Table S4. Shared and distinct genera between healthy individuals, patients with CTLA4 deficiency or combined variable immunodeficiency.

## Data Availability

All sequences, metadata files and supplementary files associated with the data analysis are available in National Center for Biotechnology Information Sequence Read Archive (Bio Project ID: PRJNA996628; https://www.ncbi.nlm.nih.gov/sra/PRJNA996628).

## Abbreviations

ASV: Amplicon sequence variants
AUROC: Area under receiver operating characteristic curve
BM: Bowel movement
CAI: Clinical activity index
CCI: Center for Chronic Immunodeficiency
CRP: C-reactive protein
CNS: Central nervous system
CTLA4: Cytotoxic T-lymphocyte antigen 4
CTLA4-D: Cytotoxic T-lymphocyte antigen 4 deficiency
CVID: Common variable immunodeficiency
FOB: Fecal occult blood
GI: Gastrointestinal
HCT: Hematopoietic cell transplantation
IBD: Inflammatory bowel disease
IEI: Inborn errors of immunity
IRB: Institutional Review Board
LC: Liquid chromatography
MS: Mass spectrometry
LDA: Linear discriminant analysis
LEfSe: Linear discriminant analysis effect size
mTOR: Mammalian target of rapamycin
NIH: National Institutes of Health
NIAID: National Institute of Allergy and Infectious Diseases
NRS: Numeric rating scale
OTU: Operational taxonomic units
P-SCCAI: Patient Simple Clinical Colitis Activity Index
PCoA: Principal coordinate analyses
QIIME2: Quantitative Insights Into Microbial Ecology version 2
SD: Standard deviation
SIBDQ: Short Inflammatory Bowel Disease Questionnaire
T_reg_: Regulatory T-cell

## References

[1] Linsley PS, Greene JL, Tan P, Bradshaw J, Ledbetter JA, Anasetti C, Damle NK. Coexpression and functional cooperation of CTLA-4 and CD28 on activated T lymphocytes. J Exp Med. 1992;176(6):1595–604. 1334116 10.1084/jem.176.6.1595 PMC2119471

[2] Krummel MF, Allison JP. CD28 and CTLA-4 have opposing effects on the response of T cells to stimulation. J Exp Med. 1995;182(2):459–65. 7543139 10.1084/jem.182.2.459 PMC2192127

[3] Wang J, Liu L, Ma J, Sun F, Zhao Z, Gu M. Common variants on cytotoxic T lymphocyte antigen-4 polymorphisms contributes to type 1 diabetes susceptibility: evidence based on 58 studies. PLoS ONE. 2014;9(1): e85982. 24465825 10.1371/journal.pone.0085982 PMC3900458

[4] Braun J, Donner H, Siegmund T, Walfish PG, Usadel KH, Badenhoop K. CTLA-4 promoter variants in patients with Graves’ disease and Hashimoto’s thyroiditis. Tissue Antigens. 1998;51(5):563–6. 9672157 10.1111/j.1399-0039.1998.tb02993.x

[5] Hunt KA, McGovern DP, Kumar PJ, Ghosh S, Travis SP, Walters JR, Jewell DP, Playford RJ, van Heel DA. A common CTLA4 haplotype associated with coeliac disease. Eur J Hum Genet. 2005;13(4):440–4. 15657618 10.1038/sj.ejhg.5201357

[6] Kuehn HS, Ouyang W, Lo B, Deenick EK, Niemela JE, Avery DT, Schickel JN, Tran DQ, Stoddard J, Zhang Y, et al. Immune dysregulation in human subjects with heterozygous germline mutations in CTLA4. Science. 2014;345(6204):1623–7. 25213377 10.1126/science.1255904 PMC4371526

[7] Schubert D, Bode C, Kenefeck R, Hou TZ, Wing JB, Kennedy A, Bulashevska A, Petersen BS, Schaffer AA, Gruning BA, et al. Autosomal dominant immune dysregulation syndrome in humans with CTLA4 mutations. Nat Med. 2014;20(12):1410–6. 25329329 10.1038/nm.3746 PMC4668597

[8] Zaremehrjardi F, Baniadam L, Seif F, Arshi S, Bemanian MH, Shokri S, Rezaeifar A, Fallahpour M, Nabavi M. A patient with CTLA-4 haploinsufficiency with multiple autoimmune presentations: a case report. Iran J Immunol. 2020;17(3):244–9. 32996901 10.22034/iji.2020.85641.1721

[9] Schwab C, Gabrysch A, Olbrich P, Patino V, Warnatz K, Wolff D, Hoshino A, Kobayashi M, Imai K, Takagi M, et al. Phenotype, penetrance, and treatment of 133 cytotoxic T-lymphocyte antigen 4-insufficient subjects. J Allergy Clin Immunol. 2018;142(6):1932–46. 29729943 10.1016/j.jaci.2018.02.055 PMC6215742

[10] Lo B, Fritz JM, Su HC, Uzel G, Jordan MB, Lenardo MJ. CHAI and LATAIE: new genetic diseases of CTLA-4 checkpoint insufficiency. Blood. 2016;128(8):1037–42. 27418640 10.1182/blood-2016-04-712612 PMC5000841

[11] Slatter MA, Engelhardt KR, Burroughs LM, Arkwright PD, Nademi Z, Skoda-Smith S, Hagin D, Kennedy A, Barge D, Flood T, et al. Hematopoietic stem cell transplantation for CTLA4 deficiency. J Allergy Clin Immunol. 2016;138(2):615-619 e611. 27102614 10.1016/j.jaci.2016.01.045

[12] Makadia P, Srinath A, Madan-Khetarpal S, McGuire M, Infante E, Zhang J, Felgar RE, Davis AW, Chong HJ, Windreich RM. Aplastic anemia and cytotoxic T lymphocyte antigen-4 haploinsufficiency treated with bone marrow transplantation. J Allergy Clin Immunol Pract. 2017;5(5):1445-1447.e1442. 28499781 10.1016/j.jaip.2017.03.007

[13] Hodi FS, O’Day SJ, McDermott DF, Weber RW, Sosman JA, Haanen JB, Gonzalez R, Robert C, Schadendorf D, Hassel JC, et al. Improved survival with ipilimumab in patients with metastatic melanoma. N Engl J Med. 2010;363(8):711–23. 20525992 10.1056/NEJMoa1003466 PMC3549297

[14] Friedman CF, Proverbs-Singh TA, Postow MA. Treatment of the immune-related adverse effects of immune checkpoint inhibitors: a review. JAMA Oncol. 2016;2(10):1346–53. 27367787 10.1001/jamaoncol.2016.1051

[15] Geuking MB, Cahenzli J, Lawson MA, Ng DC, Slack E, Hapfelmeier S, McCoy KD, Macpherson AJ. Intestinal bacterial colonization induces mutualistic regulatory T cell responses. Immunity. 2011;34(5):794–806. 21596591 10.1016/j.immuni.2011.03.021

[16] Vetizou M, Pitt JM, Daillere R, Lepage P, Waldschmitt N, Flament C, Rusakiewicz S, Routy B, Roberti MP, Duong CP, et al. Anticancer immunotherapy by CTLA-4 blockade relies on the gut microbiota. Science. 2015;350(6264):1079–84. 26541610 10.1126/science.aad1329 PMC4721659

[17] Sivan A, Corrales L, Hubert N, Williams JB, Aquino-Michaels K, Earley ZM, Benyamin FW, Lei YM, Jabri B, Alegre ML, et al. Commensal bifidobacterium promotes antitumor immunity and facilitates anti-PD-L1 efficacy. Science. 2015;350(6264):1084–9. 26541606 10.1126/science.aac4255 PMC4873287

[18] Coutzac C, Jouniaux JM, Paci A, Schmidt J, Mallardo D, Seck A, Asvatourian V, Cassard L, Saulnier P, Lacroix L, et al. Systemic short chain fatty acids limit antitumor effect of CTLA-4 blockade in hosts with cancer. Nat Commun. 2020;11(1):2168. 32358520 10.1038/s41467-020-16079-x PMC7195489

[19] Mager LF, Burkhard R, Pett N, Cooke NCA, Brown K, Ramay H, Paik S, Stagg J, Groves RA, Gallo M, et al. Microbiome-derived inosine modulates response to checkpoint inhibitor immunotherapy. Science. 2020;369(6510):1481–9. 32792462 10.1126/science.abc3421

[20] Surti B, Spiegel B, Ippoliti A, Vasiliauskas EA, Simpson P, Shih DQ, Targan SR, McGovern DP, Melmed GY. Assessing health status in inflammatory bowel disease using a novel single-item numeric rating scale. Dig Dis Sci. 2013;58(5):1313–21. 23250673 10.1007/s10620-012-2500-1 PMC4161217

[21] Irvine EJ, Zhou Q, Thompson AK. The Short Inflammatory Bowel Disease Questionnaire: a quality of life instrument for community physicians managing inflammatory bowel disease. CCRPT investigators. Canadian Crohn’s Relapse Prevention Trial. Am J Gastroenterol. 1996;91(8):1571–8. 8759664

[22] Bennebroek Evertsz F, Nieuwkerk PT, Stokkers PC, Ponsioen CY, Bockting CL, Sanderman R, Sprangers MA. The patient simple clinical colitis activity index (P-SCCAI) can detect ulcerative colitis (UC) disease activity in remission: a comparison of the P-SCCAI with clinician-based SCCAI and biological markers. J Crohns Colitis. 2013;7(11):890–900. 23269224 10.1016/j.crohns.2012.11.007

[23] Fadrosh DW, Ma B, Gajer P, Sengamalay N, Ott S, Brotman RM, Ravel J. An improved dual-indexing approach for multiplexed 16S rRNA gene sequencing on the Illumina MiSeq platform. Microbiome. 2014;2(1): 6. 24558975 10.1186/2049-2618-2-6 PMC3940169

[24] Pacold ME, Brimacombe KR, Chan SH, Rohde JM, Lewis CA, Swier LJ, Possemato R, Chen WW, Sullivan LB, Fiske BP, et al. A PHGDH inhibitor reveals coordination of serine synthesis and one-carbon unit fate. Nat Chem Biol. 2016;12(6):452–8. 27110680 10.1038/nchembio.2070 PMC4871733

[25] Simon-Manso Y, Lowenthal MS, Kilpatrick LE, Sampson ML, Telu KH, Rudnick PA, Mallard WG, Bearden DW, Schock TB, Tchekhovskoi DV, et al. Metabolite profiling of a NIST Standard Reference Material for human plasma (SRM 1950): GC-MS, LC-MS, NMR, and clinical laboratory analyses, libraries, and web-based resources. Anal Chem. 2013;85(24):11725–31. 24147600 10.1021/ac402503m

[26] Simon-Manso Y, Marupaka R, Yan X, Liang Y, Telu KH, Mirokhin Y, Stein SE. Mass spectrometry fingerprints of small-molecule metabolites in biofluids: building a spectral library of recurrent spectra for urine analysis. Anal Chem. 2019;91(18):12021–9. 31424920 10.1021/acs.analchem.9b02977 PMC6839828

[27] Smith CA, O’Maille G, Want EJ, Qin C, Trauger SA, Brandon TR, Custodio DE, Abagyan R, Siuzdak G. METLIN: a metabolite mass spectral database. Ther Drug Monit. 2005;27(6):747–51. 16404815 10.1097/01.ftd.0000179845.53213.39

[28] Wang X, Cho JH, Poudel S, Li Y, Jones DR, Shaw TI, Tan H, Xie B, Peng J. JUMPm: a tool for large-scale identification of metabolites in untargeted metabolomics. Metabolites. 2020;10(5):190. 32408578 10.3390/metabo10050190 PMC7281133

[29] Wang X, Jones DR, Shaw TI, Cho JH, Wang Y, Tan H, Xie B, Zhou S, Li Y, Peng J. Target-decoy-based false discovery rate estimation for large-scale metabolite identification. J Proteome Res. 2018;17(7):2328–34. 29790753 10.1021/acs.jproteome.8b00019 PMC6252074

[30] Callahan BJ, McMurdie PJ, Rosen MJ, Han AW, Johnson AJ, Holmes SP. DADA2: high-resolution sample inference from Illumina amplicon data. Nat Methods. 2016;13(7):581–3. 27214047 10.1038/nmeth.3869 PMC4927377

[31] McMurdie PJ, Holmes S. Phyloseq: a bioconductor package for handling and analysis of high-throughput phylogenetic sequence data. Pac Symp Biocomput. 2012:235–46. PMC3357092 22174279

[32] Varet H, Brillet-Gueguen L, Coppee JY, Dillies MA. SARTools: a DESeq2- and EdgeR-based R pipeline for comprehensive differential analysis of RNA-seq data. PLoS ONE. 2016;11(6): e0157022. 27280887 10.1371/journal.pone.0157022 PMC4900645

[33] Foster ZS, Sharpton TJ, Grunwald NJ. Metacoder: an R package for visualization and manipulation of community taxonomic diversity data. PLoS Comput Biol. 2017;13(2): e1005404. 28222096 10.1371/journal.pcbi.1005404 PMC5340466

[34] Segata N, Izard J, Waldron L, Gevers D, Miropolsky L, Garrett WS, Huttenhower C. Metagenomic biomarker discovery and explanation. Genome Biol. 2011;12(6): R60. 21702898 10.1186/gb-2011-12-6-r60 PMC3218848

[35] Chong J, Liu P, Zhou G, Xia J. Using microbiomeAnalyst for comprehensive statistical, functional, and meta-analysis of microbiome data. Nat Protoc. 2020;15(3):799–821. 31942082 10.1038/s41596-019-0264-1

[36] Langille MG, Zaneveld J, Caporaso JG, McDonald D, Knights D, Reyes JA, Clemente JC, Burkepile DE, Vega Thurber RL, Knight R, et al. Predictive functional profiling of microbial communities using 16S rRNA marker gene sequences. Nat Biotechnol. 2013;31(9):814–21. 23975157 10.1038/nbt.2676 PMC3819121

[37] Lu Y, Zhou G, Ewald J, Pang Z, Shiri T, Xia J. MicrobiomeAnalyst 2.0: comprehensive statistical, functional and integrative analysis of microbiome data. Nucleic Acids Res. 2023;51(W1):W310–8. 37166960 10.1093/nar/gkad407 PMC10320150

[38] Virtanen P, Gommers R, Oliphant TE, Haberland M, Reddy T, Cournapeau D, Burovski E, Peterson P, Weckesser W, Bright J, et al. SciPy 1.0: fundamental algorithms for scientific computing in Python. Nat Methods. 2020;17(3):261–72. 32015543 10.1038/s41592-019-0686-2 PMC7056644

[39] Jones E, Oliphant T, Peterson P. SciPy: open source scientific tools for Python. 2001. http://www.scipy.org/.

[40] Love MI, Huber W, Anders S. Moderated estimation of fold change and dispersion for RNA-seq data with DESeq2. Genome Biol. 2014;15(12):550. 25516281 10.1186/s13059-014-0550-8 PMC4302049

[41] Pang Z, Chong J, Li S, Xia J. MetaboAnalystR 3.0: toward an optimized workflow for global metabolomics. Metabolites. 2020;10(5):186. 32392884 10.3390/metabo10050186 PMC7281575

[42] Breiman L. Random forests. Mach Learn. 2001;45(1):5–32.

[43] Liu Y, Tran DQ, Lindsey JW, Rhoads JM. The association of gut microbiota and Treg dysfunction in autoimmune diseases. Adv Exp Med Biol. 2021;1278:191–203. 33523449 10.1007/978-981-15-6407-9_10 PMC9290759

[44] Postow MA, Sidlow R, Hellmann MD. Immune-related adverse events associated with immune checkpoint blockade. N Engl J Med. 2018;378(2):158–68. 29320654 10.1056/NEJMra1703481

[45] Berman D, Parker SM, Siegel J, Chasalow SD, Weber J, Galbraith S, Targan SR, Wang HL. Blockade of cytotoxic T-lymphocyte antigen-4 by ipilimumab results in dysregulation of gastrointestinal immunity in patients with advanced melanoma. Cancer Immun. 2010;10:11. 21090563 PMC2999944

[46] Tivol EA, Borriello F, Schweitzer AN, Lynch WP, Bluestone JA, Sharpe AH. Loss of CTLA-4 leads to massive lymphoproliferation and fatal multiorgan tissue destruction, revealing a critical negative regulatory role of CTLA-4. Immunity. 1995;3(5):541–7. 7584144 10.1016/1074-7613(95)90125-6

[47] Waterhouse P, Penninger JM, Timms E, Wakeham A, Shahinian A, Lee KP, Thompson CB, Griesser H, Mak TW. Lymphoproliferative disorders with early lethality in mice deficient in Ctla-4. Science. 1995;270(5238):985–8. 7481803 10.1126/science.270.5238.985

[48] Bjork JR, Bolte LA, Maltez Thomas A, Lee KA, Rossi N, Wind TT, Smit LM, Armanini F, Asnicar F, Blanco-Miguez A, et al. Longitudinal gut microbiome changes in immune checkpoint blockade-treated advanced melanoma. Nat Med. 2024;30(3):785–96. 38365950 10.1038/s41591-024-02803-3 PMC10957474

[49] Gunjur A, Shao Y, Rozday T, Klein O, Mu A, Haak BW, Markman B, Kee D, Carlino MS, Underhill C, et al. A gut microbial signature for combination immune checkpoint blockade across cancer types. Nat Med. 2024;30(3):797–809. 38429524 10.1038/s41591-024-02823-z PMC10957475

[50] Liu R, Zou Y, Wang WQ, Chen JH, Zhang L, Feng J, Yin JY, Mao XY, Li Q, Luo ZY, et al. Gut microbial structural variation associates with immune checkpoint inhibitor response. Nat Commun. 2023;14(1):7421. 37973916 10.1038/s41467-023-42997-7 PMC10654443

[51] Lee KA, Thomas AM, Bolte LA, Bjork JR, de Ruijter LK, Armanini F, Asnicar F, Blanco-Miguez A, Board R, Calbet-Llopart N, et al. Cross-cohort gut microbiome associations with immune checkpoint inhibitor response in advanced melanoma. Nat Med. 2022;28(3):535–44. 35228751 10.1038/s41591-022-01695-5 PMC8938272

[52] Davar D, Dzutsev AK, McCulloch JA, Rodrigues RR, Chauvin JM, Morrison RM, Deblasio RN, Menna C, Ding Q, Pagliano O, et al. Fecal microbiota transplant overcomes resistance to anti-PD-1 therapy in melanoma patients. Science. 2021;371(6529):595–602. 33542131 10.1126/science.abf3363 PMC8097968

[53] Baruch EN, Youngster I, Ben-Betzalel G, Ortenberg R, Lahat A, Katz L, Adler K, Dick-Necula D, Raskin S, Bloch N, et al. Fecal microbiota transplant promotes response in immunotherapy-refractory melanoma patients. Science. 2021;371(6529):602–9. 33303685 10.1126/science.abb5920

[54] Dubin K, Callahan MK, Ren B, Khanin R, Viale A, Ling L, No D, Gobourne A, Littmann E, Huttenhower C, et al. Intestinal microbiome analyses identify melanoma patients at risk for checkpoint-blockade-induced colitis. Nat Commun. 2016;7: 10391. 26837003 10.1038/ncomms10391 PMC4740747

[55] Liu T, Xiong Q, Li L, Hu Y. Intestinal microbiota predicts lung cancer patients at risk of immune-related diarrhea. Immunotherapy. 2019;11(5):385–96. 30693820 10.2217/imt-2018-0144

[56] Sakai K, Sakurai T, De Velasco MA, Nagai T, Chikugo T, Ueshima K, Kura Y, Takahama T, Hayashi H, Nakagawa K, et al. Intestinal microbiota and gene expression reveal similarity and dissimilarity between immune-mediated colitis and ulcerative colitis. Front Oncol. 2021;11: 763468. 34778085 10.3389/fonc.2021.763468 PMC8578892

[57] McCulloch JA, Davar D, Rodrigues RR, Badger JH, Fang JR, Cole AM, Balaji AK, Vetizou M, Prescott SM, Fernandes MR, et al. Intestinal microbiota signatures of clinical response and immune-related adverse events in melanoma patients treated with anti-PD-1. Nat Med. 2022;28(3):545–56. 35228752 10.1038/s41591-022-01698-2 PMC10246505

[58] Wang F, Yin Q, Chen L, Davis MM. Bifidobacterium can mitigate intestinal immunopathology in the context of CTLA-4 blockade. Proc Natl Acad Sci U S A. 2018;115(1):157–61. 29255057 10.1073/pnas.1712901115 PMC5776803

[59] Sun S, Luo L, Liang W, Yin Q, Guo J, Rush AM, Lv Z, Liang Q, Fischbach MA, Sonnenburg JL, et al. Bifidobacterium alters the gut microbiota and modulates the functional metabolism of T regulatory cells in the context of immune checkpoint blockade. Proc Natl Acad Sci U S A. 2020;117(44):27509–15. 33077598 10.1073/pnas.1921223117 PMC7959554

[60] Wang T, Zheng N, Luo Q, Jiang L, He B, Yuan X, Shen L. Probiotics lactobacillus reuteri abrogates immune checkpoint blockade-associated colitis by inhibiting group 3 innate lymphoid cells. Front Immunol. 2019;10: 1235. 31214189 10.3389/fimmu.2019.01235 PMC6558076

[61] Fasanello MK, Robillard KT, Boland PM, Bain AJ, Kanehira K. Use of fecal microbial transplantation for immune checkpoint inhibitor colitis. ACG Case Rep J. 2020;7(4): e00360. 32548190 10.14309/crj.0000000000000360 PMC7224717

[62] Chen M, Liu M, Li C, Peng S, Li Y, Xu X, Sun M, Sun X. Fecal microbiota transplantation effectively cures a patient with severe bleeding immune checkpoint inhibitor-associated colitis and a short review. Front Oncol. 2022;12: 913217. 35756645 10.3389/fonc.2022.913217 PMC9229182

[63] Wang Y, Wiesnoski DH, Helmink BA, Gopalakrishnan V, Choi K, DuPont HL, Jiang ZD, Abu-Sbeih H, Sanchez CA, Chang CC, et al. Fecal microbiota transplantation for refractory immune checkpoint inhibitor-associated colitis. Nat Med. 2018;24(12):1804–8. 30420754 10.1038/s41591-018-0238-9 PMC6322556

[64] Yang J, Li Y, Wen Z, Liu W, Meng L, Huang H. Oscillospira - a candidate for the next-generation probiotics. Gut Microbes. 2021;13(1): 1987783. 34693878 10.1080/19490976.2021.1987783 PMC8547878

[65] Lima SF, Gogokhia L, Viladomiu M, Chou L, Putzel G, Jin WB, Pires S, Guo CJ, Gerardin Y, Crawford CV, et al. Transferable immunoglobulin a-coated Odoribacter splanchnicus in responders to fecal microbiota transplantation for ulcerative colitis limits colonic inflammation. Gastroenterology. 2022;162(1):166–78. 34606847 10.1053/j.gastro.2021.09.061 PMC8678328

[66] Vacca M, Celano G, Calabrese FM, Portincasa P, Gobbetti M, De Angelis M. The controversial role of human gut Lachnospiraceae. Microorganisms. 2020;8(4):573. 32326636 10.3390/microorganisms8040573 PMC7232163

[67] Hirayama M, Nishiwaki H, Hamaguchi T, Ito M, Ueyama J, Maeda T, Kashihara K, Tsuboi Y, Ohno K. Intestinal collinsella may mitigate infection and exacerbation of COVID-19 by producing ursodeoxycholate. PLoS ONE. 2021;16(11): e0260451. 34813629 10.1371/journal.pone.0260451 PMC8610263

[68] Chang SC, Shen MH, Liu CY, Pu CM, Hu JM, Huang CJ. A gut butyrate-producing bacterium Butyricicoccus pullicaecorum regulates short-chain fatty acid transporter and receptor to reduce the progression of 1,2-dimethylhydrazine-associated colorectal cancer. Oncol Lett. 2020;20(6):327. 33101496 10.3892/ol.2020.12190 PMC7577080

[69] Boesmans L, Valles-Colomer M, Wang J, Eeckhaut V, Falony G, Ducatelle R, Van Immerseel F, Raes J, Verbeke K. Butyrate producers as potential next-generation probiotics: safety assessment of the administration of Butyricicoccus pullicaecorum to healthy volunteers. mSystems. 2018;3(6):e00094. 30417112 10.1128/mSystems.00094-18 PMC6222043

[70] Zhan Z, Liu W, Pan L, Bao Y, Yan Z, Hong L. Overabundance of Veillonella parvula promotes intestinal inflammation by activating macrophages via LPS-TLR4 pathway. Cell Death Discov. 2022;8(1):251. 35523778 10.1038/s41420-022-01015-3 PMC9076897

[71] Cortez RV, Moreira LN, Padilha M, Bibas MD, Toma RK, Porta G, Taddei CR. Gut microbiome of children and adolescents with primary sclerosing cholangitis in association with ulcerative colitis. Front Immunol. 2020;11: 598152. 33613519 10.3389/fimmu.2020.598152 PMC7893080

[72] Zhang SX, Wang J, Chen JW, Zhang MX, Zhang YF, Hu FY, Lv ZQ, Gao C, Li YF, Li XF. The level of peripheral regulatory T cells is linked to changes in gut commensal microflora in patients with systemic lupus erythematosus. Ann Rheum Dis. 2021;80(11): e177. 31732515 10.1136/annrheumdis-2019-216504

[73] Zagato E, Pozzi C, Bertocchi A, Schioppa T, Saccheri F, Guglietta S, Fosso B, Melocchi L, Nizzoli G, Troisi J, et al. Endogenous murine microbiota member Faecalibaculum rodentium and its human homologue protect from intestinal tumour growth. Nat Microbiol. 2020;5(3):511–24. 31988379 10.1038/s41564-019-0649-5 PMC7048616

[74] Parada Venegas D, De la Fuente MK, Landskron G, Gonzalez MJ, Quera R, Dijkstra G, Harmsen HJM, Faber KN, Hermoso MA. Short chain fatty acids (SCFAs)-mediated gut epithelial and immune regulation and its relevance for inflammatory bowel diseases. Front Immunol. 2019;10: 277. 30915065 10.3389/fimmu.2019.00277 PMC6421268

[75] Furusawa Y, Obata Y, Fukuda S, Endo TA, Nakato G, Takahashi D, Nakanishi Y, Uetake C, Kato K, Kato T, et al. Commensal microbe-derived butyrate induces the differentiation of colonic regulatory T cells. Nature. 2013;504(7480):446–50. 24226770 10.1038/nature12721

[76] Chudan S, Ishibashi R, Nishikawa M, Tabuchi Y, Nagai Y, Ikushiro S, Furusawa Y. Effect of soluble oat fiber on intestinal microenvironment and TNBS-induced colitis. Food Funct. 2023;14(4):2188–99. 36756938 10.1039/d2fo03396h

[77] Bui TPN, Manneras-Holm L, Puschmann R, Wu H, Troise AD, Nijsse B, Boeren S, Backhed F, Fiedler D, deVos WM. Conversion of dietary inositol into propionate and acetate by commensal Anaerostipes associates with host health. Nat Commun. 2021;12(1):4798. 34376656 10.1038/s41467-021-25081-w PMC8355322

[78] Pellicciotta M, Rigoni R, Falcone EL, Holland SM, Villa A, Cassani B. The microbiome and immunodeficiencies: lessons from rare diseases. J Autoimmun. 2019;98:132–48. 30704941 10.1016/j.jaut.2019.01.008

[79] Hajjar J, Voigt A, Conner M, Swennes A, Fowler S, Calarge C, Mendonca D, Armstrong D, Chang CY, Walter J et al. Common variable immunodeficiency patient fecal microbiota transplant recapitulates gut dysbiosis. Res Sq [Preprint]. 2023:rs.3.rs-2640584.

[80] Wang W, Zou W. Amino acids and their transporters in T cell immunity and cancer therapy. Mol Cell. 2020;80(3):384–95. 32997964 10.1016/j.molcel.2020.09.006 PMC7655528

[81] van den Bogert B, Meijerink M, Zoetendal EG, Wells JM, Kleerebezem M. Immunomodulatory properties of Streptococcus and Veillonella isolates from the human small intestine microbiota. PLoS ONE. 2014;9(12): e114277. 25479553 10.1371/journal.pone.0114277 PMC4257559

[82] Egland PG, Palmer RJ Jr, Kolenbrander PE. Interspecies communication in Streptococcus gordonii-Veillonella atypica biofilms: signaling in flow conditions requires juxtaposition. Proc Natl Acad Sci U S A. 2004;101(48):16917–22. 15546975 10.1073/pnas.0407457101 PMC534724

[83] Nedumgottil BM. Relative presence of Streptococcus mutans, Veillonella atypica, and Granulicatella adiacens in biofilm of complete dentures. J Indian Prosthodont Soc. 2018;18(1):24–8. 29430138 10.4103/jips.jips_183_17 PMC5799964

[84] Krasinski SD, Russell RM, Furie BC, Kruger SF, Jacques PF, Furie B. The prevalence of vitamin K deficiency in chronic gastrointestinal disorders. Am J Clin Nutr. 1985;41(3):639–43. 3976564 10.1093/ajcn/41.3.639

[85] Hu S, Ma Y, Xiong K, Wang Y, Liu Y, Sun Y, Yang Y, Ma A. Ameliorating effects of vitamin K2 on dextran sulfate sodium-induced ulcerative colitis in mice. Int J Mol Sci. 2023;24(3):2986. 10.3390/ijms24032986 PMC9917520 36769323

