# Correction: The intestinal microbiome and metabolome discern disease severity in cytotoxic T-lymphocyte-associated protein 4 deficiency

**DOI:** 10.1186/s40168-025-02069-y

**Published:** 2025-03-15

**Authors:** Prabha Chandrasekaran, Máté Krausz, Yu Han, Noriko Mitsuiki, Annemarie Gabrysch, Christina Nöltner, Michele Proietti, Theo Heller, Caroline Grou, Virginie Calderon, Poorani Subramanian, Drew R. Jones, Yik Siu, Clayton Deming, Sean Conlan, Steven M. Holland, Julia A. Segre, Gulbu Uzel, Bodo Grimbacher, Emilia Liana Falcone

**Affiliations:** 1 Laboratory of Clinical Investigation, National Institute On Aging (NIA), Baltimore, MD USA; 2 Center for Chronic Immunodeficiency (CCI), Medical Center, University of Freiburg, Freiburg, Germany; 3 Department of Rheumatology and Clinical Immunology, Medical Center-University of Freiburg, Freiburg, Germany; 4 Faculty of Biology, Albert-Ludwigs-University of Freiburg, Freiburg, Germany; 5 Laboratory of Clinical Immunology and Microbiology, National Institute of Allergy and Infectious Diseases (NIAID), National Institutes of Health (NIH), Bethesda, MD USA; 6 Division of Molecular Genetics and Pathology, Center for Devices and Radiological Health, Food and Drug Administration (FDA), Silver Spring, MD USA; 7 Clinic Department of Rheumatology and Immunology, Hannover Medical School, Hanover, Germany; 8 Translational Hepatology Section, National Institute of Diabetes and Digestive and Kidney Diseases (NIDDK), National Institutes of Health (NIH), Bethesda, MD USA; 9 Bioinformatics Core, Montreal Clinical Research Institute (IRCM), Montreal, QC Canada; 10 Bioinformatics and Computational Biosciences Branch (BCBB), Office of Cyber Infrastructure and Computational Biology (OCICB), National Institute of Allergy and Infectious Diseases (NIAID), National Institutes of Health (NIH), Bethesda, MD USA; 11 Metabolomics Laboratory, New York University Langone, New York, NY USA; 12 National Human Genome Research Institute (NHGRI), National Institutes of Health (NIH), Bethesda, MD USA; 13 DZIF — German Center for Infection Research, Satellite Center, Freiburg, Germany; 14 CIBSS — Centre for Integrative Biological Signaling Studies, Albert-Ludwigs-University of Freiburg, Freiburg, Germany; 15 RESIST — Cluster of Excellence, Hannover Medical School, Satellite Center Freiburg, Freiburg, Germany; 16 Center for Immunity, Inflammation and Infectious Diseases, Montreal Clinical Research Institute (IRCM), Montreal, QC Canada; 17 Department of Medicine, Université de Montréal, Montreal, QC Canada; 18 Department of Microbiology, Infectious Diseases and Immunology, Université de Montréal, Montreal, QC Canada

**Correction** **:** **Microbiome 13, 51 (2025)**

**https://doi.org/10.1186/s40168-025-02028-7**

In the Abstract, Conclusions section for this article [1], “targets” was inadvertently truncated by the Production Team as “ta”.

The original article has been updated.
