# Supplementary material for: The intestinal microbiome and metabolome discern disease severity in cytotoxic T-lymphocyte-associated protein 4 deficiency
Source: Microbiome. 2025 Feb 11;13:51. doi: 10.1186/s40168-025-02028-7 (PMC11817180; doi:10.1186/s40168-025-02028-7)
Supplement: Supplementary file 2 — Supplementary Material 1: Figure S1. Correlations between clinical parameters associated with gastrointestinal (GI) manifestations in patients with CTLA4 deficiency. Correlogram for clinical parameters in patients with CTLA4 deficiency. Circle values=coefficient of correlation (r value); circle size=strength of significance (red=positive correlation, blue=negative correlation, blank=no significant correlation). All presented r values have p<0.05. Figure S2. Alterations in phylum and genus abundances in patients with CTLA4 deficiency from NIH and CCI cohorts. (A) Heatmap of components of the core microbiome at the genus level that are detected in high fractions in CTLA4 deficiency groups (20% of the sample prevalence cut-off) (yellow = low prevalence, purple = high prevalence) (A1 = NIH cohort; A2 = CCI cohort). The generalized linear models (GLM) to find associations between microbial features and CTLA4 deficiency identified the phyla (B) and genera (C) that are significantly different in CTLA4 deficiency groups compared to healthy individuals. All comparisons for the genera are significant with p<0.05, unless a p-value is shown. Figure S3. Phylum- and genus-level differences in CTLA4 deficiency in the NIH cohort. Comparisons are provided for groups of patients with CTLA4 deficiency from the NIH cohort with different degrees of disease severity (Healthy n=16, Mild n=7, Severe No GI n= 6, Severe GI n= 19). (A) Box and violin plots indicating phylum abundances in each group. The name of the phylum is indicated in the top of each panel with the p-values for each comparison shown in the graph. Wherever the p-value is <0.05, the significance is marked with an asterisk (* = p<0.05, **=p<0.01, ***p<0.001). (B) Heat trees depicting the significant differential abundances (p<0.05) of bacterial genera between patients with CTLA4 deficiency with Severe versus Mild disease, and (C) Mild disease versus Healthy (red = higher abundance; blue = lower abundance). Figure S4. Distinct [file 40168_2025_2028_MOESM1_ESM.pdf]

Fig. S1

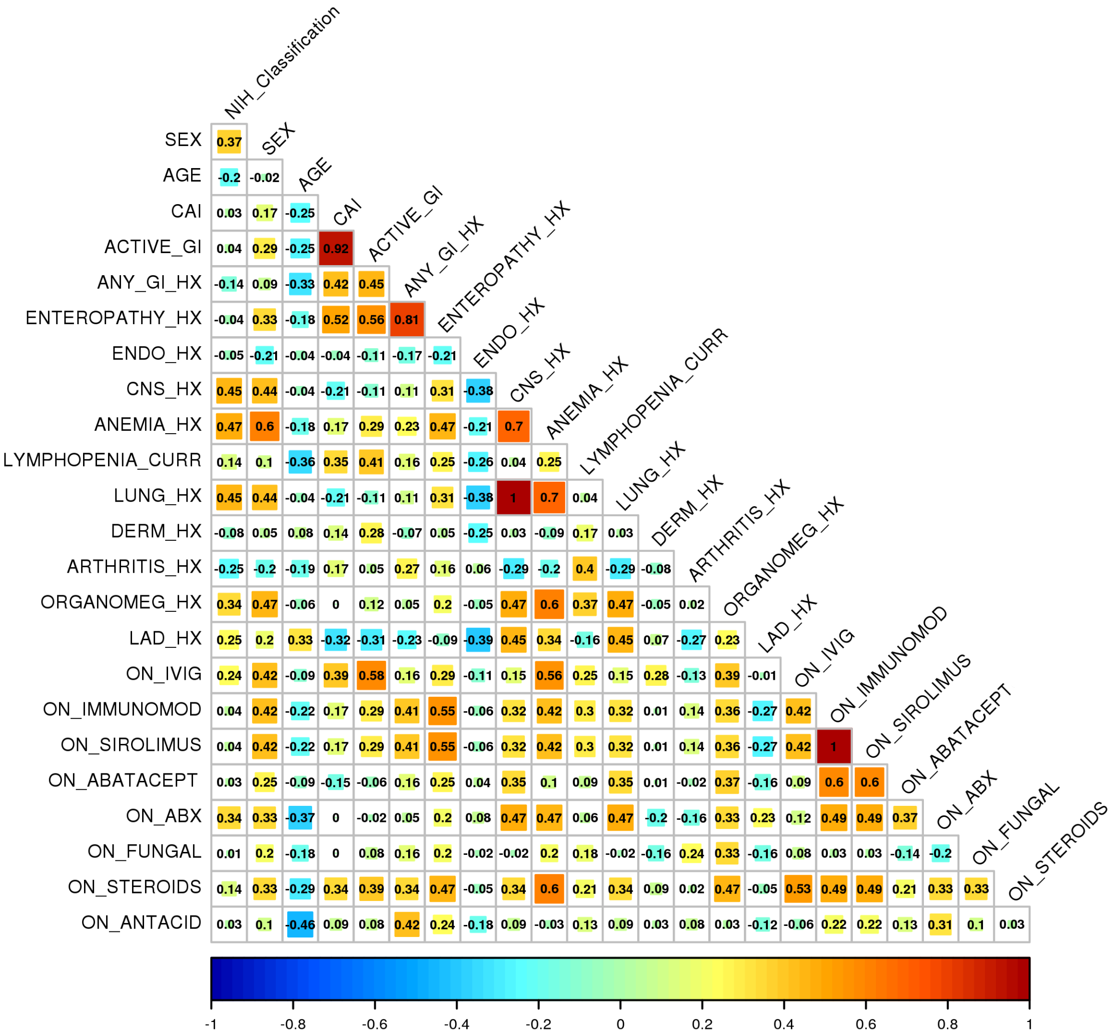
NIH_Classification
SEX
AGE
CAI
ACTIVE_GI
ANY_GI_HX
ENTEROPATHY_HX
ENDO_HX
CNS_HX
ANEMIA_HX
LYMPHOPENIA_CURR
LUNG_HX
DERM_HX
ARTHRITIS_HX
ORGANOMEG_HX
LAD_HX
ON_IVIG
ON_IMMUNOMOD
ON_SIROLIMUS
ON_ABATACEPT
ON_ABX
ON_FUNGAL
ON_STEROIDS
ON_ANTACID
-1
-0.8
-0.6
-0.4
-0.2
0
0.2
0.4
0.6
0.8
1

Fig. S2

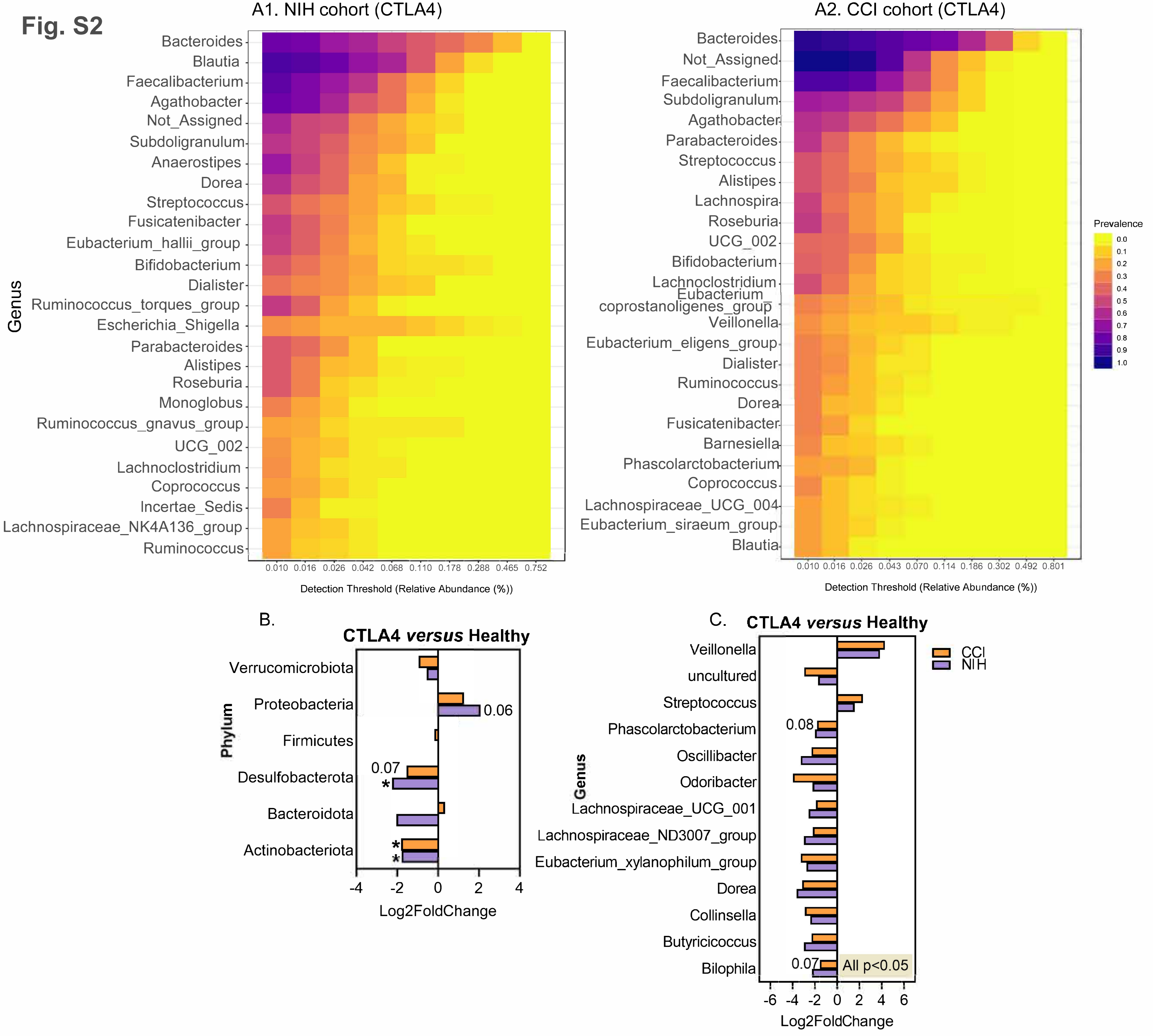
A1. NIH cohort (CTLA4)
Genus
Bacteroides
Blautia
Faecalibacterium
Agathobacter
Not_Assigned
Subdoligranulum
Anaerostipes
Dorea
Streptococcus
Fusicatenibacter
Eubacterium_hallii_group
Bifidobacterium
Dialister
Ruminococcus_torques_group
Escherichia_Shigella
Parabacteroides
Alistipes
Roseburia
Monoglobus
Ruminococcus_gnavus_group
UCG_002
Lachnoclostridium
Coprococcus
Incertae_Sedis
Lachnospiraceae_NK4A136_group
Ruminococcus
0.010 0.016 0.026 0.042 0.068 0.110 0.178 0.288 0.465 0.752
Detection Threshold (Relative Abundance (%))
A2. CCI cohort (CTLA4)
Bacteroides
Not_Assigned
Faecalibacterium
Subdoligranulum
Agathobacter
Parabacteroides
Streptococcus
Alistipes
Lachnospira
Roseburia
UCG_002
Bifidobacterium
Lachnoclostridium
Eubacterium_coprostanoligenes_group
Veillonella
Eubacterium_eligens_group
Dialister
Ruminococcus
Dorea
Fusicatenibacter
Barnesiella
Phascolarctobacterium
Coprococcus
Lachnospiraceae_UCG_004
Eubacterium_siraeum_group
Blautia
0.010 0.016 0.026 0.043 0.070 0.114 0.186 0.302 0.492 0.801
Detection Threshold (Relative Abundance (%))
Prevalence
0.0
0.1
0.2
0.3
0.4
0.5
0.6
0.7
0.8
0.9
1.0
B.
CTLA4 *versus* Healthy
Phylum
Verrucomicrobiota
Proteobacteria
Firmicutes
Desulfobacterota
Bacteroidota
Actinobacteriota
0.06
0.07
*
*
*
-4 -2 0 2 4
Log2FoldChange
C.
CTLA4 *versus* Healthy
CCI
NIH
Genus
Veillonella
uncultured
Streptococcus
Phascolarctobacterium
Oscillibacter
Odoribacter
Lachnospiraceae_UCG_001
Lachnospiraceae_ND3007_group
Eubacterium_xylanophilum_group
Dorea
Collinsella
Butyricicoccus
Bilophila
0.08
0.07
All p<0.05
-6 -4 -2 0 2 4 6
Log2FoldChange

Fig. S3

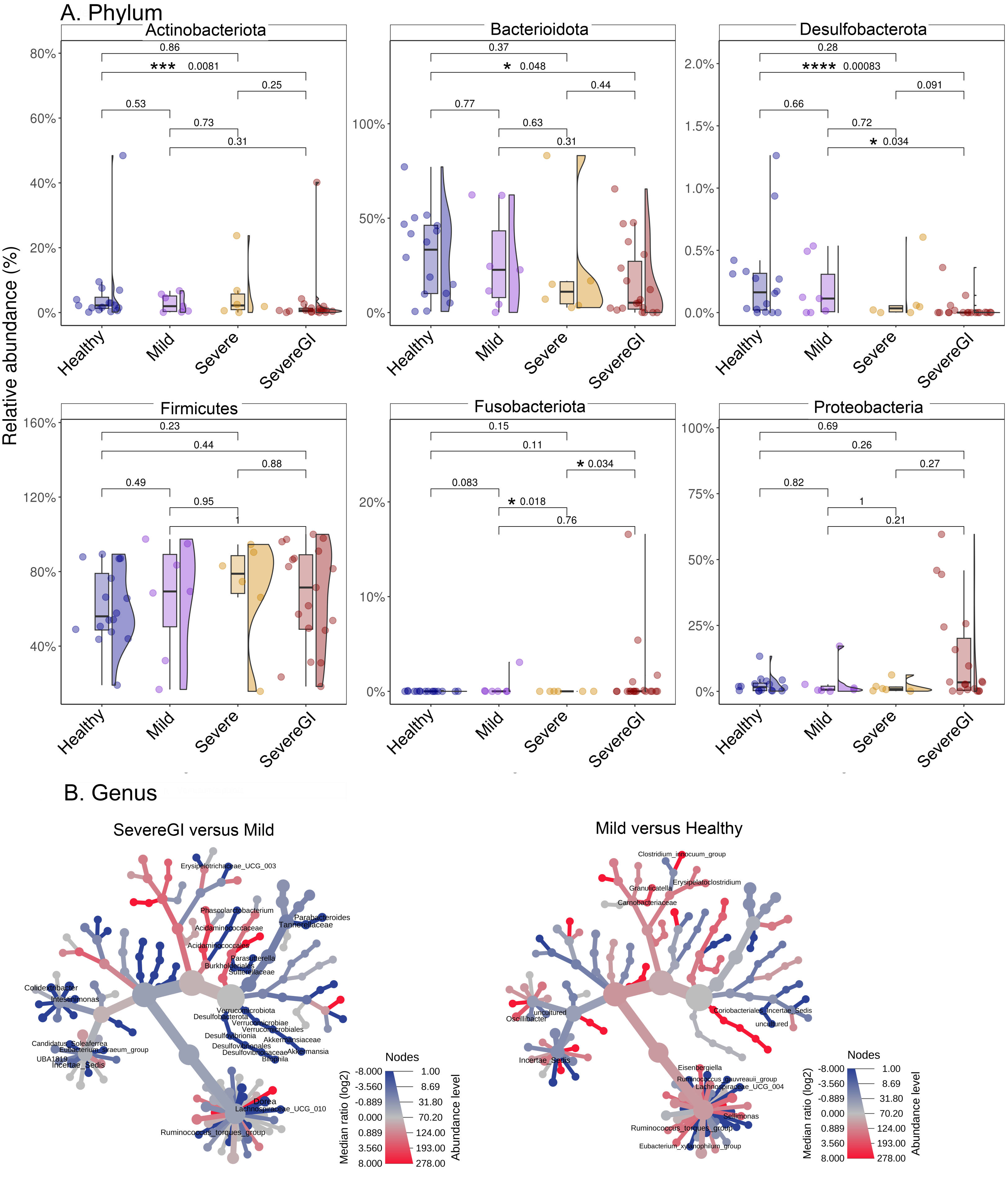
A. Phylum
Relative abundance (%)
Actinobacteriota
0.86
*** 0.0081
0.25
0.53
0.73
0.31
Bacterioidota
0.37
* 0.048
0.44
0.77
0.63
0.31
Desulfobacterota
0.28
**** 0.00083
0.091
0.66
0.72
* 0.034
Firmicutes
0.23
0.44
0.88
0.49
0.95
1
Fusobacteriota
0.15
0.11
* 0.034
0.083
* 0.018
0.76
Proteobacteria
0.69
0.26
0.27
0.82
1
0.21
Healthy
Mild
Severe
SevereGI
B. Genus
SevereGI versus Mild
Mild versus Healthy
Nodes
Median ratio (log2)
Abundance level
-8.000
-3.560
-0.889
0.000
0.889
3.560
8.000
1.00
8.69
31.80
70.20
124.00
193.00
278.00
Erysipelotrichaceae_UCG_003
Phascolarctobacterium
Acidaminococcaceae
Acidaminococcales
Parabacteroides
Tannerellaceae
Parasutterella
Burkholderiales
Sutterellaceae
Colidextribacter
Intestinimonas
Verrucomicrobiota
Desulfobacterota
Verrucomicrobiae
Verrucomicrobiales
Desulfovibrionia
Akkermansiaceae
Desulfovibrionales
Desulfovibrionaceae
Akkermansia
Bilophila
Candidatus_Soleaferrea
Eubacterium_fissicatena_group
UBA1819
Incertae_Sedis
Dorea
Lachnospiraceae_UCG_010
Ruminococcus_torques_group
Clostridium_innocuum_group
Granulicatella
Erysipelatoclostridium
Carnobacteriaceae
uncultured
Oscillibacter
Coriobacteriales_Incertae_Sedis
Eisenbergiella
Ruminococcus_gauvreauii_group
Lachnospiraceae_UCG_004
Sellimonas
Eubacterium_xylanophilum_group

Fig. S4

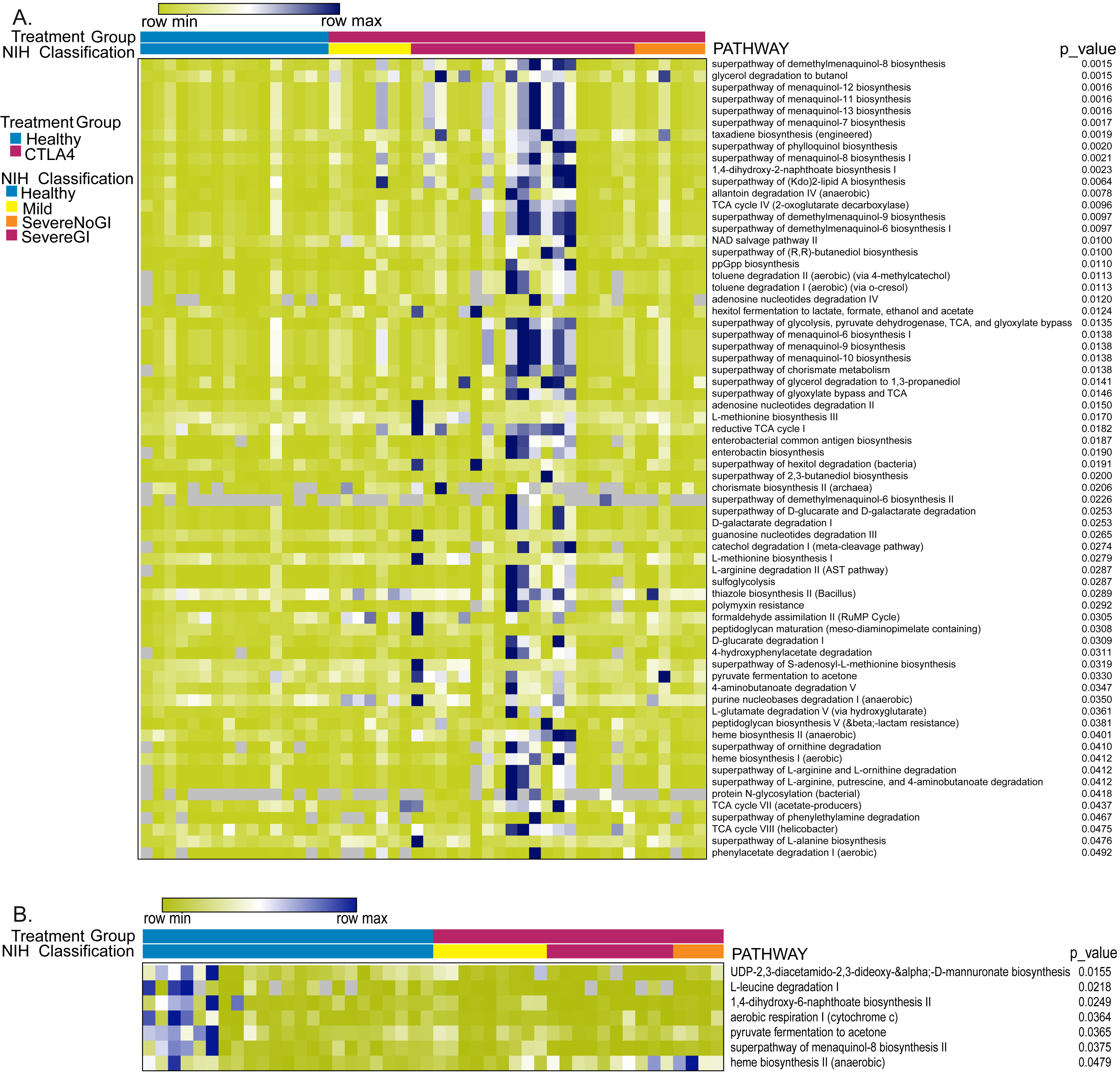
A.
row min
row max
Treatment Group
NIH Classification
PATHWAY
p_value
Treatment Group
Healthy
CTLA4
NIH Classification
Healthy
Mild
SevereNoGI
SevereGI
superpathway of demethylmenaquinol-8 biosynthesis 0.0015
glycerol degradation to butanol 0.0015
superpathway of menaquinol-12 biosynthesis 0.0016
superpathway of menaquinol-11 biosynthesis 0.0016
superpathway of menaquinol-13 biosynthesis 0.0016
superpathway of menaquinol-7 biosynthesis 0.0017
taxadiene biosynthesis (engineered) 0.0019
superpathway of phylloquinol biosynthesis 0.0020
superpathway of menaquinol-8 biosynthesis I 0.0021
1,4-dihydroxy-2-naphthoate biosynthesis I 0.0023
superpathway of (Kdo)2-lipid A biosynthesis 0.0064
allantoin degradation IV (anaerobic) 0.0078
TCA cycle IV (2-oxoglutarate decarboxylase) 0.0096
superpathway of demethylmenaquinol-9 biosynthesis 0.0097
superpathway of demethylmenaquinol-6 biosynthesis I 0.0097
NAD salvage pathway II 0.0100
superpathway of (R,R)-butanediol biosynthesis 0.0100
ppGpp biosynthesis 0.0110
toluene degradation II (aerobic) (via 4-methylcatechol) 0.0113
toluene degradation I (aerobic) (via o-cresol) 0.0113
adenosine nucleotides degradation IV 0.0120
hexitol fermentation to lactate, formate, ethanol and acetate 0.0124
superpathway of glycolysis, pyruvate dehydrogenase, TCA, and glyoxylate bypass 0.0135
superpathway of menaquinol-6 biosynthesis I 0.0138
superpathway of menaquinol-9 biosynthesis 0.0138
superpathway of menaquinol-10 biosynthesis 0.0138
superpathway of chorismate metabolism 0.0138
superpathway of glycerol degradation to 1,3-propanediol 0.0141
superpathway of glyoxylate bypass and TCA 0.0146
adenosine nucleotides degradation II 0.0150
L-methionine biosynthesis III 0.0170
reductive TCA cycle I 0.0182
enterobacterial common antigen biosynthesis 0.0187
enterobactin biosynthesis 0.0190
superpathway of hexitol degradation (bacteria) 0.0191
superpathway of 2,3-butanediol biosynthesis 0.0200
chorismate biosynthesis II (archaea) 0.0206
superpathway of demethylmenaquinol-6 biosynthesis II 0.0226
superpathway of D-glucarate and D-galactarate degradation 0.0253
D-galactarate degradation I 0.0253
guanosine nucleotides degradation III 0.0265
catechol degradation I (meta-cleavage pathway) 0.0274
L-methionine biosynthesis I 0.0279
L-arginine degradation II (AST pathway) 0.0287
sulfoglycolysis 0.0287
thiazole biosynthesis II (Bacillus) 0.0289
polymyxin resistance 0.0292
formaldehyde assimilation II (RuMP Cycle) 0.0305
peptidoglycan maturation (meso-diaminopimelate containing) 0.0308
D-glucarate degradation I 0.0309
4-hydroxyphenylacetate degradation 0.0311
superpathway of S-adenosyl-L-methionine biosynthesis 0.0319
pyruvate fermentation to acetone 0.0330
4-aminobutanoate degradation V 0.0347
purine nucleobases degradation I (anaerobic) 0.0350
L-glutamate degradation V (via hydroxyglutarate) 0.0361
peptidoglycan biosynthesis V (β-lactam resistance) 0.0381
heme biosynthesis II (anaerobic) 0.0401
superpathway of ornithine degradation 0.0410
heme biosynthesis I (aerobic) 0.0412
superpathway of L-arginine and L-ornithine degradation 0.0412
superpathway of L-arginine, putrescine, and 4-aminobutanoate degradation 0.0412
protein N-glycosylation (bacterial) 0.0418
TCA cycle VII (acetate-producers) 0.0437
superpathway of phenylethylamine degradation 0.0467
TCA cycle VIII (helicobacter) 0.0475
superpathway of L-alanine biosynthesis 0.0476
phenylacetate degradation I (aerobic) 0.0492
B.
row min
row max
Treatment Group
NIH Classification
PATHWAY
p_value
UDP-2,3-diacetamido-2,3-dideoxy-α-D-mannuronate biosynthesis 0.0155
L-leucine degradation I 0.0218
1,4-dihydroxy-6-naphthoate biosynthesis II 0.0249
aerobic respiration I (cytochrome c) 0.0364
pyruvate fermentation to acetone 0.0365
superpathway of menaquinol-8 biosynthesis II 0.0375
heme biosynthesis II (anaerobic) 0.0479

Fig. S5 1. NIH cohort 2. CCI cohort

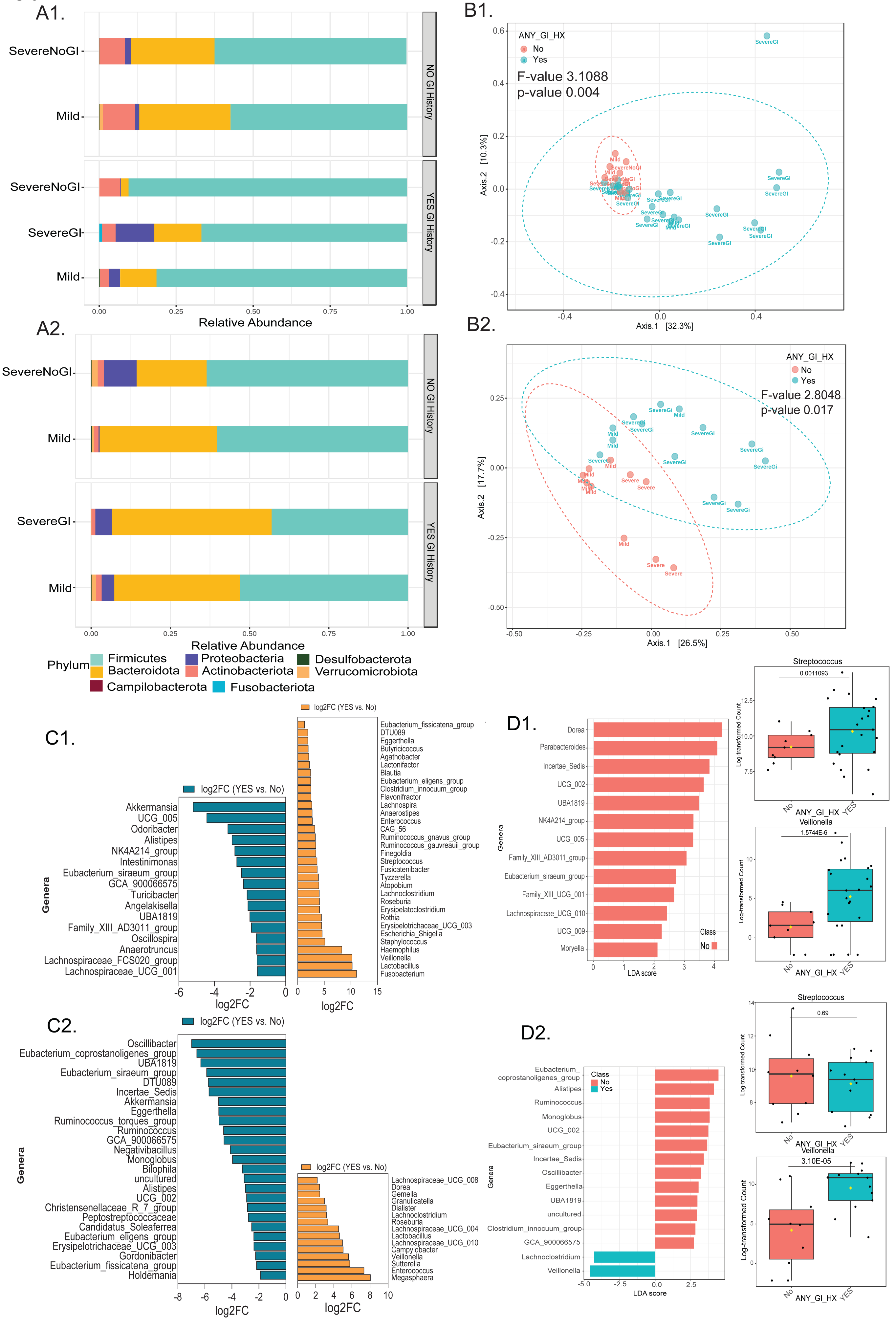

Fig. S6

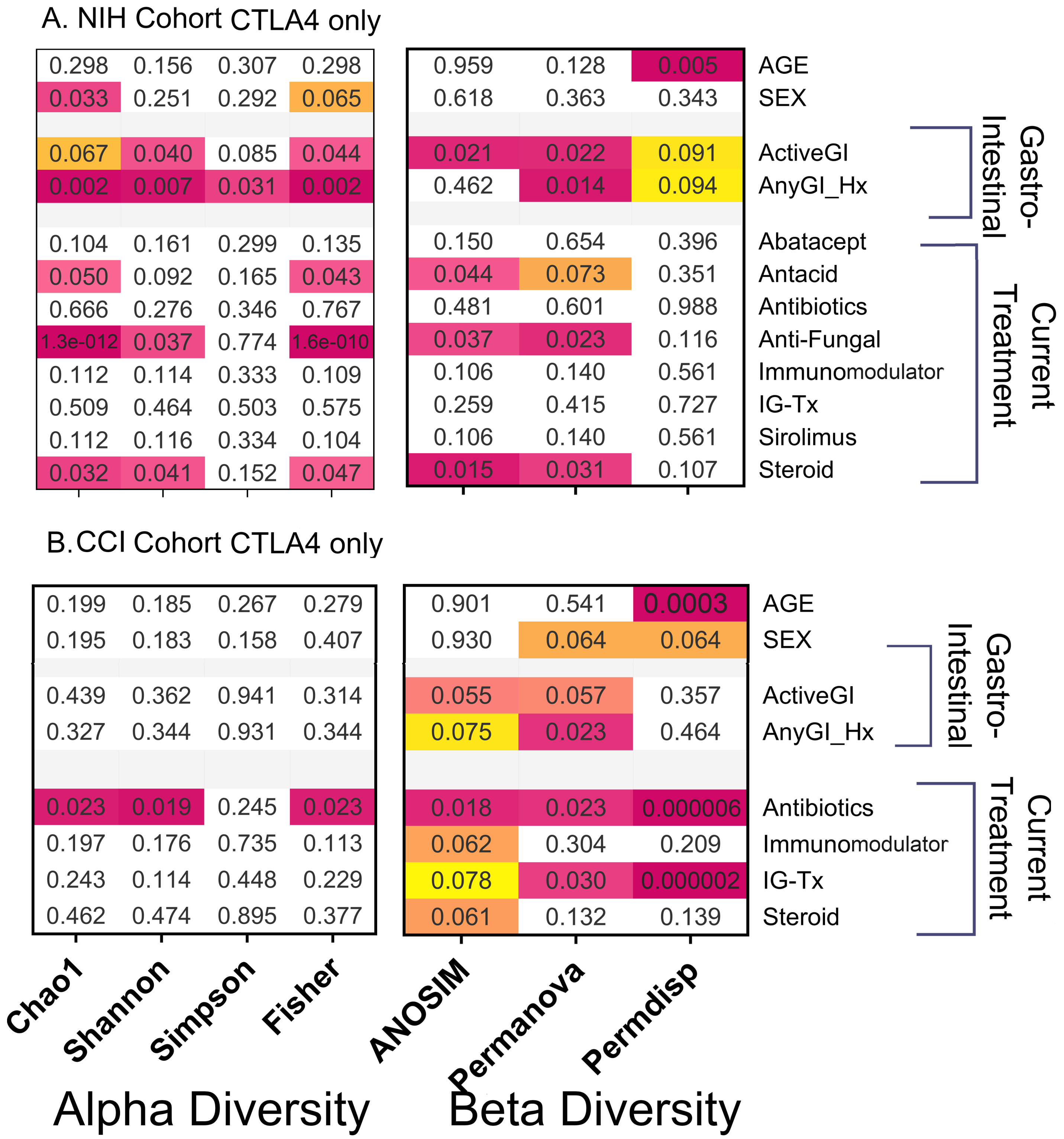
A. NIH Cohort CTLA4 only
0.298 0.156 0.307 0.298 | 0.959 0.128 0.005 AGE
0.033 0.251 0.292 0.065 | 0.618 0.363 0.343 SEX
0.067 0.040 0.085 0.044 | 0.021 0.022 0.091 ActiveGI
0.002 0.007 0.031 0.002 | 0.462 0.014 0.094 AnyGI_Hx
0.104 0.161 0.299 0.135 | 0.150 0.654 0.396 Abatacept
0.050 0.092 0.165 0.043 | 0.044 0.073 0.351 Antacid
0.666 0.276 0.346 0.767 | 0.481 0.601 0.988 Antibiotics
1.3e-012 0.037 0.774 1.6e-010 | 0.037 0.023 0.116 Anti-Fungal
0.112 0.114 0.333 0.109 | 0.106 0.140 0.561 Immunomodulator
0.509 0.464 0.503 0.575 | 0.259 0.415 0.727 IG-Tx
0.112 0.116 0.334 0.104 | 0.106 0.140 0.561 Sirolimus
0.032 0.041 0.152 0.047 | 0.015 0.031 0.107 Steroid
Gastro-Intestinal
Current Treatment
B.CCI Cohort CTLA4 only
0.199 0.185 0.267 0.279 | 0.901 0.541 0.0003 AGE
0.195 0.183 0.158 0.407 | 0.930 0.064 0.064 SEX
0.439 0.362 0.941 0.314 | 0.055 0.057 0.357 ActiveGI
0.327 0.344 0.931 0.344 | 0.075 0.023 0.464 AnyGI_Hx
0.023 0.019 0.245 0.023 | 0.018 0.023 0.000006 Antibiotics
0.197 0.176 0.735 0.113 | 0.062 0.304 0.209 Immunomodulator
0.243 0.114 0.448 0.229 | 0.078 0.030 0.000002 IG-Tx
0.462 0.474 0.895 0.377 | 0.061 0.132 0.139 Steroid
Gastro-Intestinal
Current Treatment
Chao1
Shannon
Simpson
Fisher
ANOSIM
Permanova
Permdisp
Alpha Diversity
Beta Diversity

Fig. S7

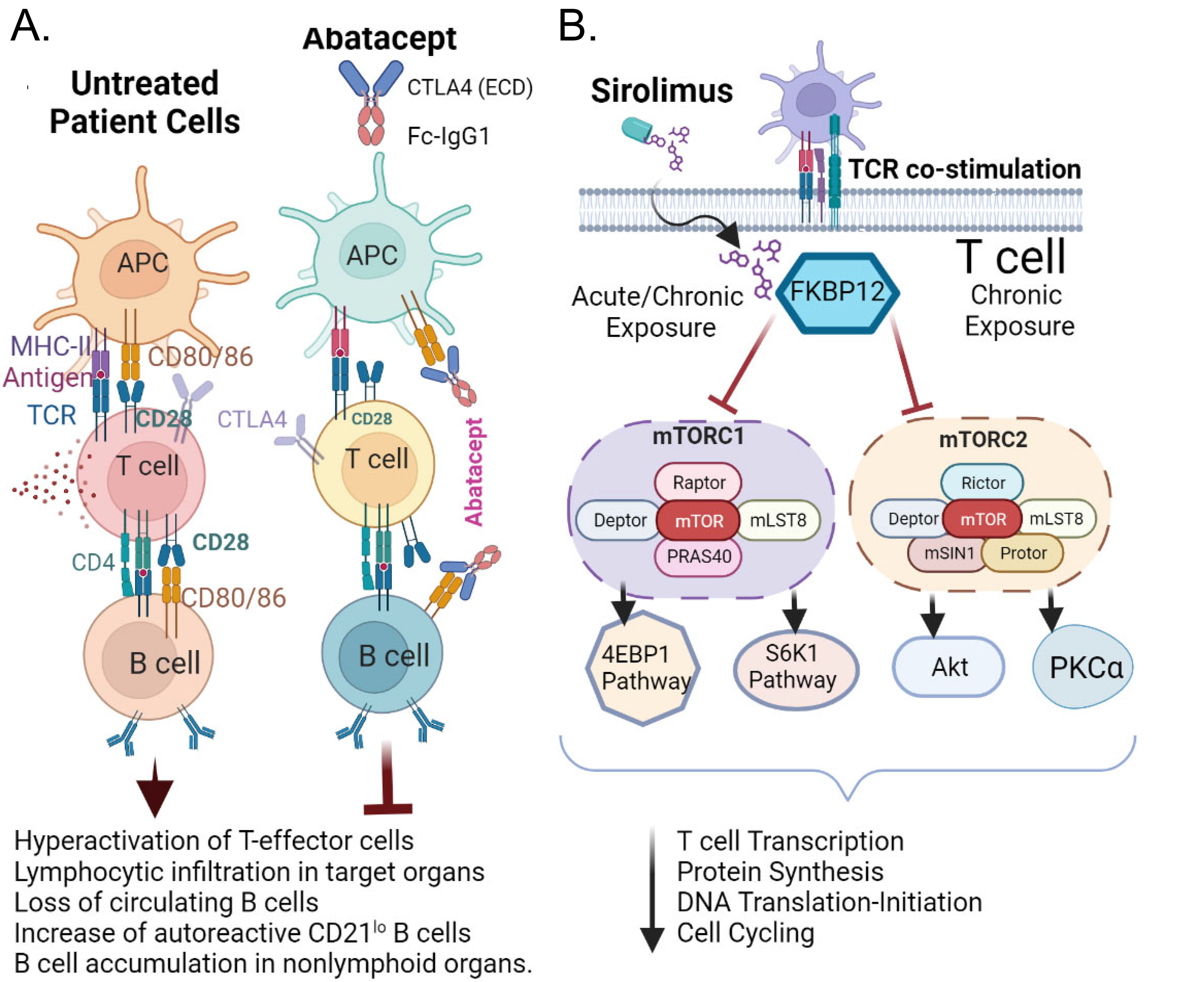
A.
Untreated
Patient Cells
Abatacept
CTLA4 (ECD)
Fc-IgG1
APC
MHC-II
Antigen
CD80/86
TCR
CD28
CTLA4
T cell
CD4
CD28
CD80/86
B cell
APC
CD28
T cell
Abatacept
B cell
Hyperactivation of T-effector cells
Lymphocytic infiltration in target organs
Loss of circulating B cells
Increase of autoreactive CD21lo B cells
B cell accumulation in nonlymphoid organs.
B.
Sirolimus
TCR co-stimulation
T cell
Acute/Chronic
Exposure
FKBP12
Chronic
Exposure
mTORC1
Raptor
Deptor
mTOR
mLST8
PRAS40
mTORC2
Rictor
Deptor
mTOR
mLST8
mSIN1
Protor
4EBP1
Pathway
S6K1
Pathway
Akt
PKCα
T cell Transcription
Protein Synthesis
DNA Translation-Initiation
Cell Cycling
