# Supplementary material for: The intestinal microbiome and metabolome discern disease severity in cytotoxic T-lymphocyte-associated protein 4 deficiency
Source: Microbiome. 2025 Feb 11;13:51. doi: 10.1186/s40168-025-02028-7 (PMC11817180; doi:10.1186/s40168-025-02028-7)
Supplement: Supplementary file 3 — Supplementary Material 2: Table S1. Demographic and clinical characteristics of participants from the NIH cohort. Table S2. Clinical scores and laboratory test results in participants from the NIH cohort. Table S3. Demographic and clinical characteristics of participants from the CCI cohort. Table S4. Shared and distinct genera between healthy individuals, patients with CTLA4 deficiency or combined variable immunodeficiency. [file 40168_2025_2028_MOESM2_ESM.docx]

**SUPPLEMENTARY TABLES**

**The intestinal microbiome and metabolome discern disease severity in cytotoxic T-lymphocyte-associated protein 4 deficiency**

Chandrasekaran, P., Krausz, M., Han, Y., … Falcone, E.L.

**Table S1. Demographic and clinical characteristics of participants from the NIH cohort.**

|  | **Healthy**  n=16 | **CTLA4-D**  n=32 |
| --- | --- | --- |
| Age, mean (SD), years | 30.5 (7.4) | 31.3 (15.9) |
| Female, n (%) | 12 (75) | 12 (37.5) |
| Race / Ethnicity, n (%)  White  Black  Latino(a) or Hispanic  Asian  Mixed  Native American | 10 (62.5)  1 (6.3)  3 (18.8)  2 (12.5)  0 (0)  0 (0) | 23 (71.9)  1 (3.1)  4 (12.5)  0 (0)  3 (9.3)  1 (3.1) |
| Disease severity (NIH classification), n (%)  None / mild  Severe without GI  Severe with GI | 16 (100)  N/A  N/A | 7 (21.9)  6 (18.8)  19 (59.4) |
| History of any GI manifestation, n (%) | 0 (0) | 23 (71.9) |
| Active GI manifestations at time of visit, n (%) | 0 (0) | 11 (34.4) |
| History of hypogammaglobulinemia, n (%) | 0 (0) | 16 (50) |
| History of any cytopenia, n (%)  Anemia  Lymphopenia  Thrombocytopenia | 0 (0) | 20 (62.5)  7 (21.9)  6 (18.8) |
| History of lymphadenopathy, n (%) | 0 (0) | 9 (28.1) |
| History of hepatosplenomegaly, n (%) | 0 (0) | 12 (37.5) |
| History of pulmonary complications, n (%) | 0 (0) | 17 (53.1) |
| History of CNS complications, n (%) | 0 (0) | 17 (53.1) |
| History of endocrinopathy, n (%) | 0 (0) | 17 (53.1) |
| History of dermatological manifestations, n (%) | 0 (0) | 9 (28.1) |
| On an antibiotic, n (%) | 0 (0) | 12 (37.5) |
| On an antifungal, n (%) | 0 (0) | 2 (6.3) |
| On immunoglobulin replacement, n (%) | 0 (0) | 11 (34.4) |
| On an immune modulator, n (%)  Any  Systemic steroids  Rituximab  Sirolimus  Abatacept | 0 (0) | 14 (43.8)  12 (37.5)  1 (3.1)  14 (43.8)  7 (21.9) |

CTLA4-D: cytotoxic T-lymphocyte associated protein 4 deficiency; NIH: National Institutes

of Health; GI: gastrointestinal.

**Table S2. Clinical scores and laboratory test results in participants from the NIH cohort.**

|  | **Normal range** | **Healthy**  n=16 | **CTLA4-D**  n=32 | ***P*-value** |
| --- | --- | --- | --- | --- |
| Body mass index (kg/m^2^, mean (SD))^1^ | 18.5-24.9 | 23 (4.2) | 24.3 (7.4) | 0.48 |
| Hg (g/dL; mean (SD)) | 13.7-17.5 | 13 (1.1) | 12.7 (2) | 0.63 |
| Hct (%; mean (SD)) | Males: 41-50  Females: 36-48 | 39.4 (4.1) | 38.6 (6.3) | 0.54 |
| Total WBC count (10^9^/L; mean (SD)) | 4.23-9.07 | 6 (2.1) | 5.7 (2.8) | 0.71 |
| Absolute lymphocyte count (10^9^/L; mean (SD)) | 1.32-3.57 | NA | 1.6 (1) | NA |
| Absolute monocyte count (10^9^/L; mean (SD)) | 0.3-0.82 | NA | 0.5 (0.2) | NA |
| Absolute neutrophil count (10^9^/L; mean (SD)) | 1.78-5.38 | NA | 3.6 (2.6) | NA |
| Platelet count (10^9^/L; mean (SD)) | 161-347 | 256 (68) | 224.5 (101.6) | 0.26 |
| CRP (mg/dL; mean (SD)) | 0-4.99 | NA | 8.9 (21.3) | NA |
| ESR (mm/hr; mean (SD)) | 0-25 | NA | 17.2 (24.1) | NA |
| Albumin (g/dL; mean (SD)) | 3.5-5.2 | NA | 4.1 (0.5) | NA |
| Fecal occult blood positive, n (%)^1^ | Negative | 0 (0) | 6 (20.7) | 0.05 |
| Fecal calprotectin >50 mcg/g, n (%)^1^ | ≤50 | 0 (0) | 8 (27.6) | 0.02 |
| Clinical activity index (CAI)^a^ >1, n (%) | 1 | 0 (0) | 11 (34.4) | 0.008 |
| Numeric rating scale, mean (SD)  ≥8, n (%) ^b, 2^ | ≥8 | 9 (1.2)  14 (100) | 6.9 (1.9)  10 (35.7) | <0.001  <0.001 |
| SIBDQ, mean (SD)  ≥56, n (%) ^c, 2^ | ≥56 | 64.5 (5.9)  12 (85.7) | 52.8 (14.4)  13 (46.2) | <0.001  0.02 |
| P-SCCAI, mean (SD)  ≤3, n (%) ^d, 2^ | ≤3 | 2 (1.2)  13 (92.9) | 5.5 (5.3)  12 (42.9) | 0.002  0.002 |

CTLA4-D: Cytotoxic T lymphocyte antigen 4 deficiency, SD: standard deviation, NA: not available, WBC: white blood cell, Hg: hemoglobin, Hct: hematocrit, platelet, CRP: C-reactive protein, erythrocyte sedimentation rate, albumin, SIBDQ: Short Inflammatory Bowel Disease Questionnaire, P-SCCAI: Patient Simple Clinical Colitis Activity Index.

^a^ CAI (clinical activity index): CAI 1= 0-2 bowel movements (BM) per day, CAI 2= 2-4 BMs per day, CAI 3= >4 BMs per day and/or presence of blood or mucus in stool and/or fistulae and/or perianal disease.

^b^ Scores equal or greater than the indicated cutoff were associated good quality of life and/or inactive disease in Surti, B., et al., 2013[20].

^c^ Scores equal or greater than the indicated cutoff were associated good quality of life and/or inactive disease in Irvine, E.J., et al., 1996[21].

^d^ Scores equal or lesser than the indicated cutoff were associated good quality of life and/or inactive disease in Bennebroek, E., et al., 2013[22].

^1^ n=29 patients with CTLA4-D

^2^ n=28 patients with CTLA4-D; n=14 healthy participants

**Table S3. Demographic and clinical characteristics of participants from the CCI cohort.**

|  | **Healthy**  n=24 | **CTLA4-D**  n=25 | **CVID**  n=20 |
| --- | --- | --- | --- |
| Age, mean (SD), years | 45.3 (18.9) | 43.3 (19) | 44.1 (13.6) |
| Female, n (%) | 8 (33.3) | 16 (64) | 12 (60) |
| Race / Ethnicity, n (%)  White  Black  Latino(a) or Hispanic  Asian  Mixed  Unknown | 20 (83.3)  0 (0)  0 (0)  4 (16.7)  0 (0)  0 (0) | 20 (80)  0 (0)  0 (0)  5 (20)  0 (0)  0 (0) | 19 (95)  0 (0)  0 (0)  1 (5)  0 (0)  0 (0) |
| Disease severity (NIH classification), n (%)  None / mild  Severe without GI  Severe with GI | 24 (100)  N/A  N/A | 11 (44)  4 (16)  10 (40) | N/A |
| History of any GI manifestation, n (%) | 0 (0) | 14 (56) | 9 (45) |
| Active GI manifestations at time of visit, n (%) | 3 (12.5) | 14 (56) | 9 (45) |
| History of hypogammaglobulinemia, n (%) | 0 (0) | 14 (56) | 9 (45) |
| History of any cytopenia, n (%) | 0 (0) | 10 (40) | 8 (40) |
| History of lymphadenopathy, n (%) | 0 (0) | 6 (24) | 7 (35) |
| History of hepatosplenomegaly, n (%) | 0 (0) | 8 (32) | 11 (55) |
| History of pulmonary complications, n (%) | 0 (0) | 10 (40) | 5 (25) |
| History of CNS complications, n (%) | 0 (0) | 7 (28) | 0 (0) |
| History of endocrinopathy, n (%) | 0 (0) | 13 (52) | 7 (35) |
| History of dermatological manifestations, n (%) | 0 (0) | 13 (52) | 1 (0.5) |
| On an antibiotic, n (%) | 0 (0) | 3 (12) | 6 (30) |
| On an antifungal, n (%) | 0 (0) | 1 (4) | 1 (5) |
| On immunoglobulin replacement, n (%) | 0 (0) | 5 (20) | 20 (100) |
| On an immune modulator, n (%)  Any  Systemic steroids  Rituximab  Sirolimus  Abatacept | 1 (4.2)  1 (4.2)  0 (0)  0 (0)  0 (0) | 6 (24)  3 (12)  1 (4)  0 (0)  0 (0) | 0 (0)  0 (0)  0 (0)  0 (0)  0 (0) |

CTLA4-D: cytotoxic T-lymphocyte-associated protein 4 deficiency; CVID: common variable immunodeficiency; CCI: Center for Chronic Immunodeficiency of the Medical Center of the Faculty of Medicine of Freiburg Treatment Consortium; GI: gastrointestinal; N/A: not applicable.

**Table S4. Shared and distinct genera between healthy individuals,** **patients with CTLA4 deficiency or combined variable immunodeficiency.**


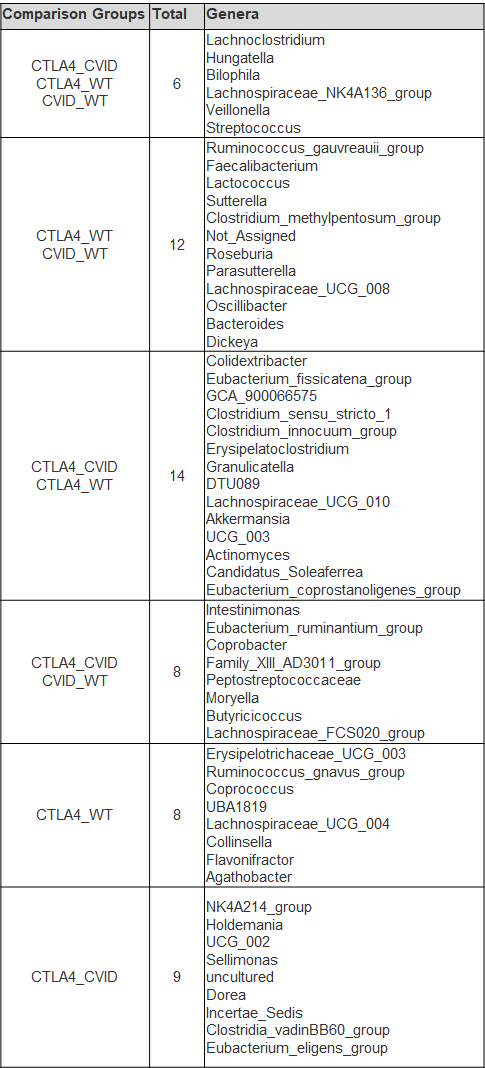


The genus list is provided for each subset of the Venn diagram presented in Figure 4D comparing the healthy (WT) group to patients with CTLA4 deficiency (CTLA4) and common variable immunodeficiency (CVID) of the CCI cohort.
